# Studies on the Presence of Mycotoxins in Biological Samples: An Overview

**DOI:** 10.3390/toxins9080251

**Published:** 2017-08-18

**Authors:** Laura Escrivá, Guillermina Font, Lara Manyes, Houda Berrada

**Affiliations:** Laboratory of Food Chemistry and Toxicology, Av. Vicent Andrés Estellés s/n, 46100 Burjassot, Spain; laura.escriva@uv.es (L.E.); guillermina.font@uv.es (G.F.); lara.manyes@uv.es (L.M.)

**Keywords:** mycotoxins, biological samples, extraction, determination, chromatography-mass spectrometry, bioaccumulation

## Abstract

Mycotoxins are fungal secondary metabolites with bioaccumulation levels leading to their carry-over into animal fluids, organs, and tissues. As a consequence, mycotoxin determination in biological samples from humans and animals has been reported worldwide. Since most mycotoxins show toxic effects at low concentrations and considering the extremely low levels present in biological samples, the application of reliable detection methods is required. This review summarizes the information regarding the studies involving mycotoxin determination in biological samples over the last 10 years. Relevant data on extraction methodology, detection techniques, sample size, limits of detection, and quantitation are presented herein. Briefly, liquid-liquid extraction followed by LC-MS/MS determination was the most common technique. The most analyzed mycotoxin was ochratoxin A, followed by zearalenone and deoxynivalenol—including their metabolites, enniatins, fumonisins, aflatoxins, T-2 and HT-2 toxins. Moreover, the studies were classified by their purpose, mainly focused on the development of analytical methodologies, mycotoxin biomonitoring, and exposure assessment. The study of tissue distribution, bioaccumulation, carry-over, persistence and transference of mycotoxins, as well as, toxicokinetics and ADME (absorption, distribution, metabolism and excretion) were other proposed goals for biological sample analysis. Finally, an overview of risk assessment was discussed.

## 1. Introduction

Mycotoxins are secondary metabolites of low molecular weight, approximately of <1000 Da, produced both pre- and post-harvest by several fungus species [1]. From about 200 identified filamentous fungi, the most prevalent toxigenic species belong to the genera *Aspergillus*, *Fusarium*, *Penicillium*, and *Alternaria*. *Fusarium* and *Alternaria* usually represent a high mycotoxicological risk at pre-harvest level or in freshly harvested products on drying, whereas *Aspergillus* and *Penicillium* toxigenic species pose a higher risk for stored food and feed products or other sorts of processing [2]. It is difficult to reduce mycotoxin exposure risks because they occur naturally under certain temperature and moisture conditions, contaminating the food throughout the food chain, in process, transport or storage [3]. The reason for mycotoxins production is not yet known since they seem not to be necessary for growth nor the development of fungi. Moreover, it is genotypically specific but not limited to one species or one toxin per species [4]. Several factors such as environmental and ecological conditions—temperature, relative humidity, substrate and use of fungicides—contribute to mycotoxin presence or production in food and feed, however, the interrelations between all these factors are not yet well understood and toxin production cannot reasonably be predicted [2,5].

***Fusarium*** genus includes over 90 described species and produces three of the most important classes of mycotoxins with respect to animal health and production; trichothecenes (TCTs), fumonisins (FBs), and zearalenones (ZONs), and the less studied emerging mycotoxins; fusaproliferin (FUS), beauvericin (BEA), enniatins (ENs), and moniliformin (MON) [6]. The toxicity of fusariotoxins varies strongly depending on the toxin and the animal species [7].

TCTs are vastly cytotoxic to eukaryotic cells since they inhibit the synthesis of nucleic acids and proteins, cell division and mitochondrial function, as well as, destabilize cell membranes. Some acute toxic events have been reported, such as alimentary toxic aleukia, characterized by gastrointestinal tract irritation, vomiting, diarrhea, leukemia, anemia, and even death [8].

FBs toxicity is mainly due to their capacity of inhibiting ceramide synthase leading to sphingolipid biosynthesis disruption with disturbances of cellular processes, such as cell growth, differentiation, morphology, permeability, and apoptosis. In addition, FB1 promotes the development of cancer in animals and seems to increase the incidence of esophageal and hepatic cancer in humans, neural tube defects, as well as, multiple diseases in experimental animals such as leukoencephalomalacia in horses and pulmonary edema syndrome in pigs [9].

ZON acute toxicity is relatively low but it strongly interferes with estrogen receptors and, as a consequence, affects the reproductive tract. Moreover, ZON leads to decreased fertility, precocious puberty, changes in weight of the thyroid, adrenal, and pituitary glands; alteration of progesterone and estradiol levels in serum, fibrosis and hyperplasia in the uterus, breast cancer, endometrial carcinoma, and liver damages that may lead to liver cancer [8].

Other secondary metabolites of potential importance not exclusively produced by *Fusarium* strains include: acuminatum, butenolide, chlamydosporol, culmorin, cyclonerodiol, equisetin, fusaprolieferins, fusarochromanones, fusaric acids, fusarins, napthoquinones, sambutoxin, and wortmannin [10].

***Penicillium*** is a large genus with 150 recognized species of which 50 or more are of common occurrence. These fungi have been reported to produce several toxins namely citrinin (CIT), cyclopiazonic acid, ochratoxin A (OTA), patulin (PAT), penicillic acid, penitrem A, roquefortine, frequentin, palitantin, mycophenolic acid, viomellein, gliotoxin, citreoviridin, and rubratoxin B [11].

OTA is mainly known for its nephrotoxic properties and it is considered to be the possible etiological cause of some kidney diseases. Moreover, OTA is mutagenic, teratogenic, neurotoxic, hepatotoxic, and immunotoxic [12].

PAT exhibits a number of toxic effects in humans and other animals, whereas CIT has antibiotic properties against Gram-positive bacteria, but it has never been used as a drug due to its high nephrotoxicity. The kidney is the major target organ of CIT toxicity, however other body parts such as liver and bone marrow have also been reported [8,11].

***Aspergillus*** genus contains significant mycotoxigenic species such as *A. flavus* and *A. parasiticus*, which make AFs; *A. ochraceus*, which makes OTA; and *A. versicolor*, which produces sterigmatocystin (STE). AFs have immunosuppressive properties and they are potent carcinogens particularly affecting the liver. They are related to hepatocellular carcinoma and several studies have linked liver cancer with the presence of AFs in food [13]. Moreover, they are associated with occasional outbreaks of acute aflatoxicosis that lead to death shortly after exposure [9].

***Alternaria*** species can produce around 70 toxic secondary metabolites which require more information about their toxicity, being of relevance; alternariol (AOH), alternariol monomethyl ether (AME), tenuazonic acid (TeA), tentoxin (TEN), and altenuene (ALT). *Alternaria* toxins are suspected mutagenic-carcinogens. TeA has been reported to be toxic to several animal species such as mice, chicken, and dogs [14]. The acute toxicity of AOH, AME, ALT, and TEN is low, although there are several reports on the mutagenic and genotoxic activities mainly of AOH and AME. These two mycotoxins are teratogenic and fetotoxic, they seem to be mutagenic, and to have estrogenic activity. AME provokes DNA strand breaks in vitro in consequence of topoisomerase poisoning, altertoxin I (ATX I) is cytotoxic and mutagenic, TeA and AME cause precancerous alterations in the esophageal mucosa of mice [15].

From the approximately 400 different compounds identified falling into the class of mycotoxins about 10–15 are considered to be of commercial interest. These are the major compounds in their families and those most commonly found. Based on the effects on human and animal health, AFs, FBs, TCTs, OTA, ZON, and PAT are recognized as the most important food mycotoxins [4]. However, the severity of the effects that mycotoxins produce largely depends on the ingested amounts, exposure duration, and toxic synergisms that may result from the simultaneous ingestion of different mycotoxins [1,8].

The International Agency for Cancer Research (IARC) has formally classified a number of mycotoxins as agents that are proven, Group 1 (AFB1, AFB2, AFG1, and AFG2); and possibly, Group 2B (OTA, FB1, and FB2, AFM1) carcinogenic to humans [16].

Besides their notorious toxicity, some mycotoxins are thermally stable and demonstrate several levels of bioaccumulation [1]. Mycotoxin occurrence in food and feed is either consequence of direct contamination of plant materials or products thereof, or by carry-over of mycotoxins and their metabolites into animal tissues, milk, and eggs after contaminated feed intake [5]. The term carry-over is often used to describe mycotoxin transfer from feed to edible tissues in order to enable a risk evaluation for the consumer arising from feeding mycotoxin-contaminated diets to food producing animals. This carry-over is usually reported as carry-over factor (transfer factor, bio-concentration factor, etc.) or as carry-over rate (transfer rate, bio-concentration rate, etc.). Both expressions of carry-over are sometimes presented as percentage of concentration or intake, respectively. As a consequence of carry-over and bioaccumulation, mycotoxin contamination was reported not only in a number of agricultural commodities, foods and feedstuffs, but also in animal derived products and biological fluids and tissues from humans and animals at geographically diverse locations [4].

With regard to bioaccumulation, some studies reported that ZON is accumulated in living organisms, being capable of contaminating all trophic levels of the food chain, from crop plants to human consumers [17]. Residues of ZON, α-zearalenol (α-ZOL), β-zearalenol (β-ZOL), and DON were detectable in pig liver, muscle, and bile after 28 days of mycotoxin feeding [18]. DON was accumulated in mice spleen, liver, lung, and kidney following similar kinetics to plasma, with maximum detectable concentrations at 15–30 min after oral exposure [19]. However, its rapid absorption, distribution, and elimination may contribute to its generally low carry-over [20]. OTA was absorbed into the body and distributed at a high concentration in the kidney, which is considered the major target organ [12]. Several studies have demonstrated transplacental transfer of OTA in swine and humans, showing OTA concentration in fetal serum to be twice the maternal concentration [21]. The ability to transform AFB1 in feed to AFM1 in milk has been examined in the past demonstrating that the extent of carry-over (2.5–5.8%) was directly correlated to milk yield in cows [22]. Low AFBs carry-over (AFM1 0.02% and AFM2 0.31%) was reported in donkey milk after naturally contaminated feed administration (AFB1: 202 and AFB2: 11 μg/kg), being not detectable after 28 h from the last contaminated feeding [23]. Negligible carry-over rates (0.0075% and 0%–0.0017%) were observed in cow milk after DON (2.62–5.24 mg/Kg) and ZON (0.33–0.66 mg/Kg) contaminated feed administration [24]. Due to the lipophilic nature of some mycotoxins, such as ENs and BEA, detectable concentrations were recovered from broiler and mice organs and tissues [25,26].

Extensive analytical efforts have been made to enable fast and reliable analysis of a large number of mycotoxins in biological samples. Due to mycotoxins, general prevention and control strategies involve very low concentration limits and the application of effective, sensitive, and accurate methods for their detection is required. *Liquid-liquid extraction (LLE)* by aqueous and/or organic solvents is used largely depending on mycotoxin structure. Additional energy may increase the LLE efficiency in *ultrasound energy (UE)* or *microwave-assisted extraction (MAE)* [27], while solvent boiling point increase by pressure retains the liquid phase in *pressurized liquid extraction (PLE)*, also known as *accelerated solvent extraction (ASE)* [28]. *Dispersive liquid-liquid micro extraction (DLLME)* is based on a ternary component solvent system where dispersant and extractant solvents are combined enhancing the surface area between the organic and the aqueous phase and facilitating the achievement of equilibrium state [29]. In *salting out liquid-liquid extraction (SALLE)* the addition of an inorganic salt into a miscible mixture forces the formation of a two-phase system [30], whereas *QuEChERS* (*Quick Easy Cheap Effective Rugged and Safe*), based on a modified solvent clean-up that uses extraction in ACN followed by a salting out step and a quick dispersive solid-phase extraction (d-SPE), expands the polarity range of the amenable compounds and allows extract purification by using small amounts of non-chlorinated organic solvents [1]. *Solid phase extraction* (*SPE*) is a more rapid alternative than LLE, which retains analytes on a special sorbent cartridge, often used for clean-up and extract pre-concentration after the selection of the most appropriate packing materials required to reach high and stable recovery rates [31].

Extracts from biological samples can be complicated mixtures where trace amounts of a target molecule may be masked by interfering compounds, affecting the separation resolution and the sensitivity of the results. The liquid extracts could be charged onto a large variety of sorbent materials, mainly immuno-affinity columns (IAC) consisting of immobilized antibodies with excellent recovery and specificity but high costs [4]. SPE cartridges, MycoSep columns, and MIPs are cheaper alternatives for sample purification [32].

There are a variety of selective and sensitive techniques for mycotoxin determination. *High-Performance Liquid Chromatography* (HPLC) is widespread because of its superior performance and reliability compared with *thin-layer chromatography* (TLC) with high quality of separation and low limits of detection (LOD). *Gas chromatography-mass spectrometry* (*GC-MS*) is used for determination of organic compounds with thermal stability and volatility, as well as for non-volatile mycotoxins chemically derivatized [33]. Multiple detection systems may be coupled to chromatography; fluorescence (FD), ultra-violet (UV), diode-array (DAD), electrochemical (EC), mass spectrometry (MS), and tandem-mass spectrometry (MS/MS), which has advanced in the last years to the status of the reference in the field of mycotoxin analysis. *Immunoaffinity methods* such as ELISA rely on antigen-antibody reaction, normally based on a competitive assay. Direct ELISA is quick and eliminates cross-reactivity, while indirect ELISA with higher immunoreactivity is generally more sensitive but cross-reactivity may lead to false positive results or overestimation, so further confirmation is required by another analytical method [4,5]. Recently, the advances in nano-sensor technologies for mycotoxin determination have gained considerable importance, since aptamers offered themselves to be ideal candidates as biocomponents in biosensors (aptasensors) [34].

In this study, the analytical methods for determining the presence of mycotoxins and their metabolites in biological samples over the last 10 years were discussed. Relevant data on extraction methodology, detection techniques, sample size, limits of detection, and quantitation, of most studied mycotoxins were evaluated herein. The studies were classified by both the analyzed matrix/matrices and by their main purposes for biological samples analysis. The origin of the analyzed biological samples (animal species) was also investigated. Relevant information regarding mycotoxins bioavailability, bioaccumulation, and ADME (absorption, distribution, metabolism and excretion) was collected. Finally, an overview of human risk assessment based on available biomonitoring data was discussed.

## 2. Results and Discussion

### 2.1. Mycotoxins Analysis in Biological Fluids

#### 2.1.1. Serum

One of the most common techniques for mycotoxin analysis in serum is LLE with different solvents. The single compound OTA was commonly extracted from human serum using CHCl_3_ [35,36] in combination with SPE [37] or IAC [38], or with CH_2_Cl_2_ [39,40], often followed by IAC [21]. Direct SPE [41] or IAC [42,43] techniques were also performed, even combined between them [44] for OTA extraction from human serum. ACN was used for LLE of ENs B and B1 [45], and DON [46,47] from chicken serum samples. ZON was extracted from rat serum using t-butyl methyl ether (TBME) [48,49], while AFB1 was extracted from human serum by direct IAC procedure [50].

When multi-mycotoxins were analyzed from serum, ACN was widely used for the extraction of several compounds including DON, 3-ADON, 15-ADON, DOM-1, T-2, HT-2, OTA, FB1, AFB1, ZON, α-ZOL, β-ZOL, zearalanone (ZAN), α-zearalanol (α-ZAL), β-zearalanol (β-ZAL), and EN A, A1, B, B1 from pig [51,52,53,54], laying hens, chicken, and turkey poults serum [55,56,57]. OTA and OTα extraction from human serum was performed by the mixture CHCl_3_/isopropanol [58], while DLLME approach using the solvents mixture ACN/EtOAc was developed for the extraction of AFs, OTA, FUS-X, STG, FBs, ENs, and BEA from fish serum [59].

Common serum sample size was 250 μL, ranging from 50 μL to 6 mL in some cases. Achieved LODs were between 0.000091 and 12 μg/L and and LOQs ranged from 0.025 to 17 μg/L.

Table 1 shows the latest studies of one single mycotoxin and multi-mycotoxins studies in serum, including sample size, studied mycotoxins, extraction and detection methods, and LODs-LOQs.

#### 2.1.2. Urine

Urinary studies often encompassed a large number of mycotoxins and metabolites, where various extraction techniques were combined to achieve the highest variety of studied compounds. High method sensitivity is of most importance since the concentration of these analytes in urine samples is often present in a very low concentration range. From the relatively few studies performed on one single mycotoxin in urine, OTA was the most common one, extracted from human urine by IAC [60,61,62], an automated multi-fiber SPME system [63], or the classical LLE with CHCl_3_-isopropanol [64].

Other compounds such as STE [65], DON-GlcA [66], and AFB1-N7-Gua [67] were individually analyzed in urine, by SPE, dilute-and-shoot, and SPE-IAC, respectively. Several authors have performed the so named “fast” sample preparation approaches such as filter-and-shoot; dilute-and-shoot; and dilute-evaporate-and-shoot techniques for multi-mycotoxin extraction in urine. Several mycotoxins including DON, DON-3-GlcA, DON-15-GlcA, DOM-1, NIV, T-2, HT-2, HT-2-4-GlcA, FB1, AFB1, AFB2, AFG1, AFG2, AFM1, FB1, FB2, ZON, ZAN, α-ZOL, β-ZOL, ZON-14-GlcA, ZAN-14-GlcA, α-ZOL-14-GlcA, β-ZOL-14-GlcA, OTA, OTα, EN B, DH-CIT, were extracted from human urine by direct dilution (1/10 factor) with H_2_O/ACN/HCOOH (94:5:1) [68,69] or H_2_O/ACN (90:10) [64,70,71,72]. The advantage of the simple sample preparation in these fast techniques needs to be compensated by the latest MS instrumentation, and highlights a high requirement for equipment with heightened sensitivity [68] or methods involving SPE or IAC cleanup. Therefore, the complexity of urine matrix and the low analyte concentrations expected in urine, lead to the consideration of more elaborated extraction techniques, and make sample clean-up often necessary. For instance, the combination of filter-and-shoot methodology and EtOAc-LLE followed by the SPE method was carried out for the extraction of 32 and 18 mycotoxins and metabolites, respectively, from human urine [73,74].

The SPE technique has been also widely used for urine mycotoxin extraction in recent years, from a single compound extraction; STG from cattle urine [65], to multi-mycotoxin studies including several mycotoxins such as DON, DON-GlcAs, DOM-1, DOM-1-GlcA, AFM1, FB1, ZON, α-ZOL, β-ZOL, ZAN, α-ZAL, β-ZAL, OTA extracted from human, rat, swine, and bovine urine using C18 SPE cartridges [75,76,77]. The extraction of ZON, α-ZOL, β-ZOL, ZAN from bovine urine was performed by TBME-LLE followed by hexane washing and C18 SPE [78].

Different sample preparation protocols were compared for the extraction of DON, OTA, FB1, AFB1, ZON, T-2, HT-2, AFB1, CIT, DOM, DON-2-GlcA, ZON-14-GlcA, α-ZOL, β-ZOL, 4-OH-OTA, OTα, AFM1, AFB1-N7-Gua from human urine, including fast dilute-and-shoot techniques, and methodologies based on LLE-SPE. Due to the low signal intensity reached by dilute-and-shoot methodologies they were considered not suitable for routine mycotoxin monitoring and SPE procedure was deemed necessary. Thus, EtOAc/formic acid (99:1) LLE followed by SAX-SPE procedure was selected after its comparison with LLE-Oasis HLB SPE cartridges clean-up [79].

In some cases, SPE extraction was combined with IAC procedure. For instance, AFM1, OTA, DON, DOM-1, α-ZOL, β-ZOL, and FB1 were extracted from human and pig urine after sample pass through an Oasis HLB column followed by IAC clean-up [80]. Sample clean-up by a multi-antibody IAC (Myco6in1) and Oasis HLB SPE connected in tandem was performed for the analysis of DON, DOM-1, OTA, AFB1, AFM1, FB1, ZON- and α-ZOL from pig urine [81]. The extraction of AFB1-N7-Gua from human urine was performed using two SPE procedures; MCX SPE and Bond elute LRC C18 SPE, intercalated by IAC clean-up procedure [67].

Direct IAC procedures were carried out for the extraction of AFB1, AFB2, AFG1, AFG2, OTA, DON, ZON, FB1, FB2, T-2, HT-2 [82,83], DON, DOM-1 [84,85,86], CIT, OH-CIT [87,88], FB1, FB2 [89], AFM1, OTA, FB1, FB2, OTA- and OTα [82] from human urine; and for ZON, ZAN, and their metabolites from bovine and swine urine [90].

QuEChERS procedure has been widely used for mycotoxin analysis in urine [91]. A similar approach based on a salting-out assisted ACN extraction followed by a dispersive solid phase extraction (d-SPE) was used for the analysis of 15 mycotoxins and metabolites including DOM-1, DON, 3-ADON, FUS-X, DAS, NIV, NEO, HT-2, T-2, ZON, α-ZOL, β-ZOL, ZAN, α-ZAL, β-ZAL from human urine [31,92,93]. SALLE methodology based on ACN/NaCl-C18 extraction was selected to analyze DON, DOM-1, 3-ADON, 15-ADON, ZON, α-ZOL, β-ZOL, ZAN, α-ZAL, β-ZAL from human urine [94]. Similar SALLE procedure performed in two steps (EtOAc-ACN) was used for the extraction of DON, NEO, AFB1, AFM1, HT-2, T-2, OTA, OTα, ZON, α-ZOL, β-ZOL, and FB1 from human and pig urine [30].

Urine sample size ranged from 100 μL to 20 mL. Achieved LODs were between 0.000125 and 12 μg/L and LOQs ranged between 0.0005 and 40 μg/L.

Table 2 and Table 3 show the latest studies of single and related mycotoxin analysis, and multi-mycotoxin determination in urine, respectively. Sample size, studied compounds, extraction and detection methods, and LODs-LOQs are included.

#### 2.1.3. Minor Biological Fluids and Fluid Combinations

Major reported fluids, such as serum and urine, were often combined and analyzed together by a single methodology. OTA and OTα were extracted by CHCl_3_/isopropanol LLE from human serum and urine samples [95,96]. Oasis HLB SPE was used for the extraction of DON, ZON and its metabolites from pig serum and urine [97], while graphitized carbon black cartridges were used for ENs analysis from human serum and urine samples after LLE with MeOH/H_2_O [98]. The extraction of CIT from human serum and urine was performed by IAC after the comparison of two clean-up methods based on C18 SPE and IAC procedures [96,99]. The combined analysis of mycotoxins from serum, urine, and feces has often been performed and may be due to the interest of these biological matrices in toxicokinetic and ADME (absorption, distribution, metabolism and excretion) studies. Thus, ENs extraction from rat samples (serum, urine, feces) was performed by LLE using ACN [100] and EtOAc [101]. DON, ZON and its metabolites were analyzed by SPE-IAC in horse urine, serum and feces samples [102], and DON, DOM, and their sufonates were extracted from excreta and intestinal content from broiler chickens, pullets, roosters, and turkeys by LLE using the mixture MeOH/H_2_O/formic acid (49.5:49.5:1) [103].

Some studies were focused on minor analyzed biological fluids such as breast milk and bile. OTA and AFM1 were analyzed in human breast milk by LLE in different stages using CHCl_3_, ACN, and petroleum ether [106,107]. AFB1, AFB2, AFG1, AFG2, AFM1, and OTA were extracted by LLE (acidified ACN-EtOAc) with low temperature purification (LTP) after the evaluation of other procedures such as LLE (CHCl_3_-NaCl, ACN) and SPE [108,109]. QuEChERS methodology was satisfactorily performed for the analysis of several mycotoxins in human breast milk including DON, 3-ADON, NIV, FUX-N, DAS, NEO, T-2, HT-2, ZON and metabolites, OTA, STG, ENs, BEA, and AFs [110]. Direct IAC was used for ZON, AFM1, and AFM2 extraction from cow [22], human [111] and donkey [23] breast milk samples. DON, DOM-1, T-2 and HT-2 were extracted from bile and serum samples from pig and chicken by LLE using MeOH/H_2_O and EtOAc [112,113].

Other biological fluids such as saliva, nasal secretions, and amniotic fluid of pregnant women have been also analyzed, but data published so far, still do not allow their use as quantitative alternative tools for assessing environmental exposures and they are often included in larger studies comprising a wide range of organs and tissues [28,114].

Table 4 shows the latest studies of mycotoxin analysis in minor biological fluids, individually or combinations of fluids, including sample size, studied compounds, extraction and detection methods, and LODs-LOQs.

### 2.2. Mycotoxin Analysis in Organs and Tissues

Many combinations of different methodologies have been carried out for mycotoxin extraction from solid biological samples namely tissues and organs. LLE techniques have been performed with several solvents; ACN and ACN-H_2_O were used for BEA and ENs extraction from mice samples including liver, kidney, colon, fat, brain, muscle, tumor, urine, and serum [26,115]. Masked and conjugated forms of DON and ZON were extracted using ACN/formic acid (99/1) from several rat samples including plasma, urine, liver, kidney, bladder, spleen, lung, stomach, small intestine, and large intestine [116]. ABF1 and OTA were extracted with the same solvent mixture from rat plasma, liver, and kidney [117]. LLE for ENs and type A trichothecenes (T-2, HT-2, DAS) was performed using EtOAc in several samples of rat and broiler, respectively [118,119].

Other solvents have been used in recent years for mycotoxin LLE from organs and tissues. For instance, OTA was extracted using CH_2_Cl_2_ [120] and by a solvent mixture of ice-cold absolute ethanol/trichloroacetic acid [121] from pig and rat samples; plasma, liver, and kidney, respectively.

TBME was used for ZON extraction from several rat samples including serum, bile, urine, lung, liver, spleen, kidneys, heart, testes, brain, muscle, adipose tissue, stomach, and small intestine [122]. In some cases LLE was followed by other extraction or purification systems. After ACN-LLE of T-2, rat liver and kidney samples were passed through a purification column (activated charcoal: celite: aluminum trioxide) for sample purification [123]. The combination of LLE followed by SPE has been widely used for multi-mycotoxin extraction from several biological samples. OTA was extracted from hen kidney, liver, and bile by CH_2_Cl_2_-LLE followed by SPE [27]. The type B trichothecenes FUS-X and NIV were extracted from pig and chicken plasma, urine, feces, liver, kidney, spleen, muscle, intestine heart, screta, and bile by the mixture ACN/H_2_O (3:1) followed by C18 Sep-pak silica cartridge [124,125], while type A trichothecenes T-2, HT-2, T-2 triol extraction from pig samples (plasma, fat, muscle, stomach, brain, small intestines, heart, lung, spleen, urine and feces) was performed by EtOAc-LLE combined with bond-elut mycotoxin SPE cartridge [126]. The emerging mycotoxins ENs and BEA were extracted using ACN followed by SPE silica column from broiler and poultry liver and tissues [127] or C18 cartridges from fish liver, viscera, tissue, and head [128]. Several trichothecenes; NIV, DON, DOM, NEO, 3-ADON, 15-ADON, T-2-triol, HT-2, and T-2 were extracted from chicken and pig muscle and liver combining ACN/EtOAc (1:3)-LLE and Oasis HLB cartridges [129].

In addition to LLE and SPE techniques, some authors included a hexane deffation step to ensure the removal of fat components present in the matrix, which could interfere in the detection process. The extraction of DON, 3-ADON, 15-ADON, and DOM-1 from chicken samples including muscle, liver, kidney, and fat, was performed by EtOAc-LLE followed by hexane deffation and Oasis HLB cartridge [130]. The extraction of ZON, ZAN and their metabolites α-ZOL, β-ZOL, α-ZAL, and β-ZAL from bovine liver and muscle was performed by several steps including MeOH and EtOAc extraction, intercalated by repeated hexane defattion steps [131]. FB1 and its derivated aminopentol-1 (AP-1) were extracted from swine liver by MeOH/H_2_O (80:20)-LLE followed by hexane deffation and Oasis HLB cartridge [132]. The extraction of 28 mycotoxins and metabolites from several animal species (dog, rabbit, rat) and human samples including urine, blood, feces, saliva, nasal secretions, breast milk, amniotic fluid of pregnant women, liver, spleen, lung, kidney, stomach, colon, brain, urine, blood, and feces was studied throughout by the comparison of three extraction methods based on LLE, QuEChERS, and PLE. Although the three methodologies showed satisfactory extraction efficiency, PLE was selected using the mixture ACN/H_2_O/acetic acid (80:19:1) for biological fluids and ACN/H_2_O/hexane/acetic acid (60:14:25:1) for organs and tissues [28]. OTA was extracted by ACN-LLE with hexane defattion followed by IAC from cow serum, liver, kidney, muscles, fat, intestine, and milk [133], while CHCl_3_/phosphoric acid (10:1)-LLE followed by IAC OchraTest WB was used for OTA extraction from muscles, liver, and kidneys from swine, cattle, sheep, horses, fish, chickens, turkeys, geese, and ducks [134]. The same solvent mixture was used to extract ZON and its metabolites from goat plasma, urine, feces, and liver followed by IAC cleanup procedure [135]. Type A trichothecenes T-2, HT-2, and T-2 triol were extracted from boar liver, kidney spleen, hearth, muscle, lung, ovary, and uterus by MeOH-LLE followed by IAC [136].

IAC technique has also been combined with previous SPE using EtOAc in ChemElut columns for the extraction of DON, DOM-1 from pig plasma, bile, urine, liver, kidney, and muscle [137]. FB1 was extracted from turkey poult plasma by ACN extraction in C18 supelclean column followed by SAX cartridge, and from muscle, liver, and kidney using ACN/MeOH (50:50) extraction, followed by hexane defattion and passed through a fumoniprep cartridge [138]. Since a wide variety of different tissues and organs were analyzed, sample size used for the analysis ranged from 25 mg to 20 g in solid samples and between 50 μL and 5 mL for liquid biological samples. Achieved LODs were in the range of 0.015–200 μg/Kg-μg/L, and LOQs ranged from 0.05–600 μg/Kg-μg/L.

Table 5 shows the latest studies of on single mycotoxin analyzed in organs and tissues, where OTA, followed by DON were the most common ones. Singles studies of ZON, NIV, FB1, T-2, and HT-2 were also performed. In Table 6 the latest studies focused on structurally related compounds carried out in organs and tissues are shown, while multi-mycotoxin studies are summarized in Table 7. The sample size, studied compounds, extraction and detection methods, and LODs-LOQs are indicated.

### 2.3. Most Common Methodologies

From the analyzed studies it was shown that LLE (24%) follow by LLE-SPE (19%) and IAC (17%) was often preferred to extract mycotoxins from biological samples in recent years. Other less used extraction techniques were dilute-and-shoot (10%), LLE-IAC (9%), SPE-IAC (9%), and SPE (6%).

In serum and other fluid samples more than 50% of the studies performed mycotoxins extraction by LLE procedures. However, mycotoxin analysis in urine included a wide variety of methods, with fast techniques such as dilute-and-shoot (31%), followed by IAC (28%), SPE-IAC (11%), and LLE-SPE (11%) being the most representative. With regard to organs and tissues analysis almost half of the studies were based on LLE-SPE (45%) followed by LLE-IAC (26%), and LLE (19%).

Regarding mycotoxin determination the great majority (55%) was performed by LC-MS/MS-including HRMS. Other detection systems such as LC-FD (23%), ELISA (8%), and GC-MS/MS (4%) were also used. It should be noted that this detection system trend remains similar when analyzing serum individually, other biological fluids, and even organs and tissues. However, the LC-MS/MS proportion considerably increases (79%) in the case of urine sample analysis. This preference by MS/MS detectors could be explained by the very low mycotoxin levels generally found in urine samples, along with the clear trend towards multi-analyte method development and application in urine mycotoxin biomarker research [104].

### 2.4. Most Studied Mycotoxins

The most analyzed mycotoxin considering all biological samples was OTA, either alone or in combination with other mycotoxin determination. Indeed, the studies focused on one single compound (or structurally related compounds) and were mainly about OTA, and in minor proportion ZON and its metabolites, DON and its metabolites, ENs and BEA, FBs, AFs, T-2, and HT-2.

With regard to serum samples, this predominance of OTA was even higher, becoming the main compound in almost half of the studies, followed by AFs, DON-ZON, and their metabolites, and the minor *Fusarium* mycotoxins ENs and BEA. As it was reported below, urine samples included the largest number of compounds in a single analysis, however, the same tendency followed in serum was shown (OTA > ZON-DON > AFs), including other commonly studied mycotoxins; FBs, T-2, and HT-2. In minor biological fluids OTA, DON, and AFs were the most analyzed mycotoxins, followed by the emerging fusarotoxins. In the case of organs and tissue analysis, the most representative mycotoxins were OTA, type A and B TCTs, and ZON including its metabolites.

### 2.5. Biological Sample Origin

**Figure 1:**
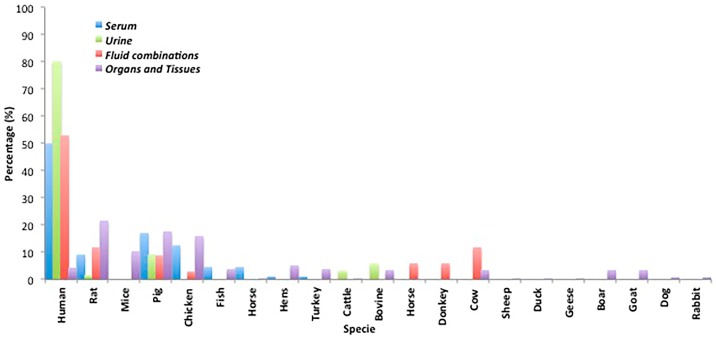
Animal species of biological sample origin: percentage of studies in serum, urine, fluids, and tissues.

The animal species of origin for the studied biological samples were analyzed. As it was expected, when non-invasive collection samples were used, human samples were interesting goals for mycotoxin determination. Thus, as it is shown in Figure 1, half of the serum samples studied as individually matrix (only serum analysis), were from human provenance (50%), followed by pig (17%), chicken (13%), rat (9%), and in minor proportion fish (5%), horse (5%), hens (1%), and turkey (1%). Similarly, in the case of fluid combination studies, including feces analysis, half of the samples were also from human (53%), followed by rat (12%), cow (12%), pig (9%), and minor proportion horse (6%), donkey (6%), and chicken (3%).

On the other hand, in studies involving mycotoxin urine analysis the great majority were from human (80%) and to a lesser extent from pig (9%), bovine (6%), cattle (3%), and rat (2%). Finally, the studies involving mycotoxins analysis in organs and tissues were generally focused on laboratory animal samples, mainly rat (22%), pig (18%), chicken (16%), mice (10%), hens (5%), and in minor proportion other animal species such as fish (4%), bovine (3%), goat (3%), boar (3%), cow (3%), dog (1%), and rabbit (1%). Due to these studies being relatively complex and which often included a wide number of different matrices, biological samples from human (4%) were also found (i.e., saliva, nasal secretions, amniotic fluid, breast milk, etc.).

### 2.6. Expected Purposes of Biological Sample Analysis

**Figure 2:**
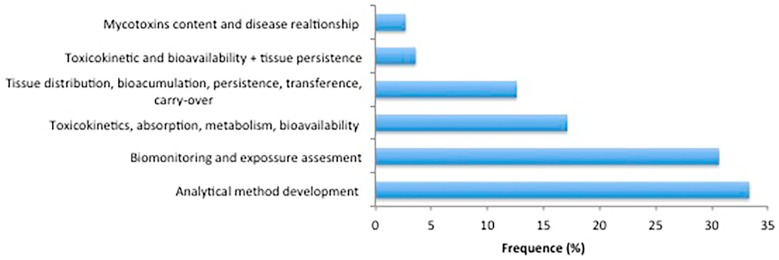
Study classification depending on the main purpose of mycotoxin determination in biological samples.

There has been shown wide importance of mycotoxin analysis in biological samples in recent years since large and varied information can be obtained from them. Thus, the studies of mycotoxins in biological samples performed in recent years had different purposes, from analytical method development—including a small method demonstration/application by analyzing a few number of samples, sometimes part of a larger pilot study—or determination of mycotoxin content and its relation with some diseases (e.g., nephropathy), to toxicokinetics, ADME (absorption, distribution, metabolism, elimination) and bioavailability studies, tissue persistence data in different animal species, as well as human biomonitoring and exposure assessment. As Figure 2 shows, the most common studies were focused on method development, due to the high sensitivity requirements for mycotoxin determination in biological samples considering the low levels generally present in them. On the other hand, human biomonitoring is increasingly being recognized as an efficient and cost-effective way of assessing human exposure to food contaminants, including mycotoxins.

**Figure 3:**
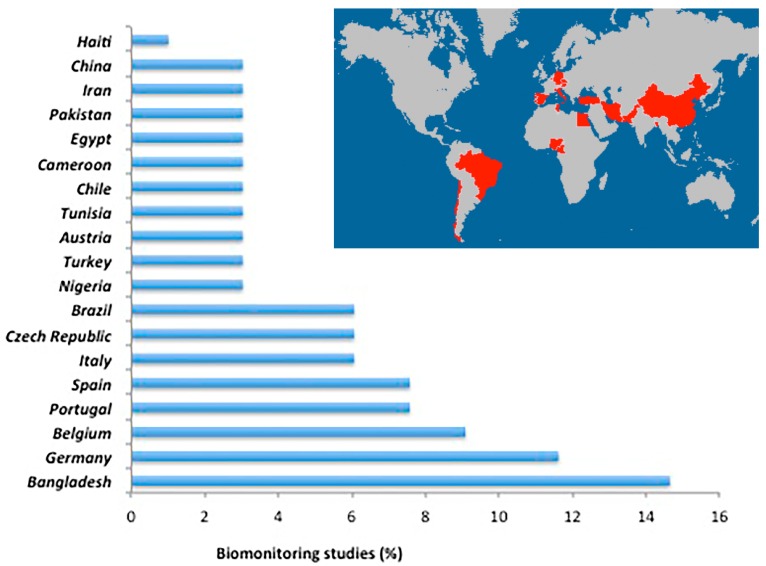
Percentage of biomonitoring and exposure assessment studies performed through mycotoxin analysis in biological samples worldwide.

Interestingly, using validated biomarkers of exposure it is possible to cover exposure from all sources, decreasing uncertainties related to occurrence and consumption rates. Moreover, it can be used to establish population reference ranges and identify vulnerable consumer groups and individuals with higher exposures [140]. For human biomonitoring easily accessible biological matrices such as urine or blood were used, with urine being preferred for several reasons including non-invasive sampling and higher acceptance by study participants. Consequently, biomonitoring studies have been frequently performed almost worldwide (Figure 3), including Nigeria [70], Bangladesh [58,69,85,86,88,96], Haiti [69], Turkey [64,106], Belgium [73,74,105], Portugal [60,61,89], Spain [36,61,93], Germany [35,62,64,68,69,86,87], Italy [77,111], Austria [72], Czech Republic [38,43], Tunisia [41], Brazil [40,109], Chile [44], Cameroon [141], Egypt [50], Pakistan [37], Iran [107], and China [84].

In these population studies, most often performed on human urine, samples from volunteers, from 27 up to 418, were collected and analyzed. Some of these studies reported additional information to that of the sample analysis. Thus, the correlation between mycotoxin content and different parameters such as the ingested diet—thought-out food questionnaires completed by the volunteers [36,68], estimation of the probably daily intake (PDI) [44,69], correlation with socio-demographic factors and anthropometric characteristics [141], or exposure to airborne molds [50] were evaluated.

With regard to studies focused on toxicokinetics, absorption, metabolism, and bioavailability, mycotoxin concentration was generally determined after mycotoxin feeding or administration; oral, per os, intravenous (IV), intraperitoneal (IP). Other studies were focused on mycotoxin tissue distribution and persistence in several animals such as chicken [125], mice [19,26,139], rat [122], pig [126]; bioaccumulation, and persistence [7,138], carry-over [20,22,24,32,133], and tissue residues [18,137].

It must be borne in mind that all this information about mycotoxin toxicokinetics, metabolism, and bioavailability is highly necessary to be both able to calculate PDIs in individuals or populations and to establish the TDIs by the regulatory authorities, and thus to enable exposure assessment to these toxic and ubiquitous compounds.

### 2.7. Mycotoxin Bioaccumulation Findings

Broad information has been obtained in the last years with regard to mycotoxins bioavailability, toxicokinetics, ADME, bioaccumulation, and tissue persistence by mycotoxin analysis in biological samples, with the main focus on DON, ZON, OTA, ENs and BEA, NIV, T-2 and FBs.

#### 2.7.1. DON and Metabolites

DON was detected in plasma (12 µg/L), liver, kidney, spleen, heart, and brain up to 19.5 µg/g after oral administration (25 mg/kg bw) in mice with highest plasma concentrations within 5–15 min after dosing [139]. Similarly, DON was reported in plasma, spleen, liver, lung and kidney after oral and intranasal administration (5 mg/kg bw) in mice with maximal concentrations within 15–30 min, declining to 75–90% after 120 min. Moreover, plasma and tissue DON concentrations were 1.5–3 times higher after intranasal exposure than following oral exposure suggesting that DON was more toxic nasally administered than orally in mice [19]. Also in pig, DON was detected in serum (5–17 µg/L), kidney, urine, bile, liver, muscle at low concentrations after 28 days feed supplementation (DON: 0.28–2.31, DON-sulfonate: 1.85 mg/kg). DON-sulfonate was stable under porcine digestive tract conditions and probably absorbed to the same extent as DON [137]. DON-3-sulfate was the major DON metabolite in chicken, pullet, rooster, and turkey after oral administration of DON by naturally contaminated feed (0.2–11 mg/kg). Fast and efficient absorption of DON between crop and jejunum was observed, followed by the conversion to DON-3 sulfate in intestinal mucosa, liver, and possibly kidney, and the rapid elimination into excreta via bile and urine [106]. DON showed low absolute oral bioavailability (19.3%) after oral administration (0.75 mg/kg bw) in broiler chickens. Volumes of distribution, total body clearance, and elimination half-life were 4.99 L/kg, 0.12 L/min kg, and 27.9 min, respectively, after IV administration [113]. Rapid clearance (t_1/2α_ = 20.4 min, t_1/2β_ = 11.8 h) was observed in mice with 5% and 2% maximum plasma DON concentrations remaining after 8 and 24 h, respectively, with DON distribution and clearance kinetics in other tissues similar to that of plasma [139]. Differences in the urinary metabolite profile of DON in human and rat were observed. DON and DON glucuronide were found in both human and rat urines, whereas DOM-1 and its glucuronide conjugate were only detected in rat urine. Human DON urinary levels ranged 0.003 and 0.008 µg/mL whereas rat DON and DOM-1 urinary levels were between 1.9 and 4.9 µg/mL and 1.6 and 5.9 µg/mL, respectively, after oral administration (3.6 mg/kw bw/day over 4 days) [76]. DON urinary daily excretion of 35.2 μg was determined in humans after 49.2 μg DON daily intake, representing 68.3% of the established DON provisional maximum tolerable daily intake (PMTDI) [31].

#### 2.7.2. ZON and Metabolites

ZON absolute oral bioavailability was 10.3% after oral administration (16 mg/kg) in rats with elimination half-life of 8.5 h. The systemic clearance, volume of distribution, and elimination half-life after IV administration (2 mg/kg) were 6.5 L/h/kg, 4.7 L/kg, and 1.9 h, respectively [48] while in broiler chickens ZON volumes of distribution, total body clearance, and elimination half-life were 22.26 L/kg, 0.48 L/min kg, and 3.9 min, respectively, after oral administration (0.3 mg/kg bw) [113]. ZON was rapidly absorbed (Tmax = 0.32–0.97 h) and eliminated (t_1/2el_ = 0.29–0.46 h) after oral and IV administration (3 mg/kg bw) in poultry, showing absolute oral bioavailability of 7–10% [55]. Accordingly, rapid absorption and low absolute oral bioavailability (2.7%) was shown in rats after oral administration (8 mg/kg). ZON was excreted unchanged in rat urine (0.5%) and bile (0.91%), showing average clearance and volume of distribution of 5.0–6.6 L/h/kg, and 2–4.7 L/kg, respectively, after IV infusion over 6 h (1.12–2.25 mg/h/kg). The highest ZON concentrations were found in small intestine, kidneys, liver, adipose tissue, and lung [122]. ZON was distributed (t_1/2α_ = 3.15 h) and eliminated (t_1/2elβ_ = 3.15 h) after single IV injection (1.2–2.4 mg/kg bw) in goat. Only α-ZOL and β-ZOL were detected in liver tissues at 48 h after IV administration. ZON, α-ZOL, and β-ZOL were excreted in urine and feces, β-ZOL being the predominant metabolite. The ZON and ZOL in urine were mostly in their glucuronide and/or sulfate conjugated forms, while those in feces were largely in their free forms [135]. ZON glucoronidation degree was 27% in pig urine (α-ZOL 88%, β-ZOL 94%) and 62% in liver (α-ZOL 77%, β-ZOL 29%). High amounts of ZON and non-glucuronidated ZOL and α-ZOL were found in muscles, indicating that ZON metabolism is not restricted to hepatic and gastrointestinal metabolic pathways [142]. ZON biotransformation to α-ZOL and β-ZOL were equally reported after IV administration in poultry, but increased for β-ZOL after oral administration indicating presystemic biotransformation [55]. Highest values of ZON carry-over factor were identified in the same tissues after oral administration bolus (150 µg/kg) and diet supplementation (50 µg/kg) in boar, showing ZON residues in spleen (20 ng/g), cardiac muscle (18 ng/g), kidneys (15 ng/g), muscle (12 ng/g), uterus (11 ng/g), and kidneys (10 ng/g) [136].

Serum and urine concentrations of DON, ZON and its metabolites increased with diet concentration increase in pig (ZON 0.01–0.29 mg/kg and DON 0.03–4.52 mg/kg; over 29 days), showing high correlation between the dietary DON intake and the sum of DON and DOM-1 concentration in serum, but accumulation was not shown. ZON, α-ZON, DON, and DOM-1 were detected in serum, urine, and liquor at lower concentrations [97]. ZON was clearly formed from ZON-14G, while the acetylated forms of 3-ADON and 15-ADON were hydrolyzed in the stomach after oral administration in rats, in contrast to DON-3G. Rats can directly glucuronidate ADONs without deacetylation, showing DON-3-GlcA accumulation in the small intestines [116]. DON and ZON residues were found in pig bile, liver, and muscle with the highest residues in bile after both organic and conventional wheat feeding [18]. Non-quantification plasma level were found for ZON and T-2 after oral administration (T-2 0.02 mg/kg bw and ZON 0.3 mg/kg bw) in broiler chickens [113]. Good correlation was observed between the amount of mycotoxins ingestion and the amount of excreted biomarkers in urine 24 h after administration in pig, showing linear dose-response (r^2^: 0.68–0.78) for the mycotoxin and its biomarker (DON-DOM-1, OTA, AFB1-AFM1, FB1, ZON-α-ZOL). Mean percentages of dietary mycotoxins excreted as biomarkers for ZON (0.6–5.7 μg/kg bw), DON (7.2–77.4 μg/kg bw), FB1 (3.7–150.2 μg/kg bw), OTA (0.2–1.3 μg/kg bw), and AFB1 (0.2–1.3 μg/kg bw) were 36.8%, 28.5%, 2.6%, 2.6%, and 2.5%, respectively [81].

#### 2.7.3. OTA

OTA was detected in 28.8% of the analyzed swine kidney samples (*n* = 1092) in concentrations ranging from 0.2 to 29.2 μg/kg, but non-quantifiable OTA levels were found in muscle, liver and kidney of cattle, sheep, horse, fish, chicken, turkey, geese, and duck [134]. Relevant OTA biliary excretion after dietary supplementation (10 and 200 μg/kg) during 6 weeks was shown in laying hens with a constant ratio between OTA bile concentration and ingested OTA. Higher levels of OTA were reported in bile than in kidney and liver [27]. However, OTA residues were not detected in cow tissues and milk, but a small amount of OTA (0.1 μg/kg) was detected in plasma after dietary OTA supplementation (>100 μg/kg) during 28 days, indicating OTA non carry-over into milk and tissues [133].

#### 2.7.4. ENs and BEA

EN B1 was rapidly absorbed (t_1/2α_ = 0.15 h, Tmax = 0.24 h), distributed and eliminated (t_1/2elα_ = 0.15 h; t_1/2elβ_ = 1.57 h) after oral administration (0.05 mg/kg/bw) in pigs with absolute oral bioavailability of 90.9% indicating clear systemic exposure, and rapid distribution and elimination (t_1/2elα_ = 0.15 h; t_1/2elβ_ = 1.13 h) after IV administration (0.05 mg/kg/bw) [53]. EN B1 and EN B were poorly absorbed after oral and IV administration (0.2 mg/kg/bw) in chicken, with absolute oral bioavailabilities of 0.05% and 0.11%, respectively. Both were quickly distributed to the tissues, with mean volumes of distribution of 33.91 and 25.09 L/kg, respectively, and mean total body clearance of 7.10 and 6.63 L/h/kg. Oxidation was the major phase I biotransformation pathway for both ENs, but neither glucuronide nor sulfate phase II metabolites were detected [45]. EN A was detected in rat serum after EN A dietary supplementation during 28 days (465 mg/kg) in an exposure time-dependent manner, reaching serum concentration of 4.76 mg/mL in the fourth week. However, EN A was not detected in feces and urine samples [101]. EN A was detected in rat tissues and fluids after 28 days feed supplementation (20.91 mg/kg bw/day) with the highest concentrations in liver (23 mg/kg), and contents of jejunum (9.6 mg/kg), colon (7.3 mg/kg), and stomach (4.6 mg/kg), as well as in serum (5 mg/kg) [118]. BEA and ENs trace levels were detected in poultry tissues with concentrations lower than 2 μg/kg [127], and both emerging mycotoxins were found in mice tissues and serum after IP administration (5 mg/kg bw, 2–3 days), with higher amounts in liver (EN B: 2.9 µg/kg and BEA: 41.7 µg/kg), and fat (EN B: 2.5 µg/kg and BEA: 33 µg/kg), indicating their tendency to bio-accumulate in lipophilic tissues [26]. Moreover, BEA and ENs crossed the blood-brain barrier in mice exerting a high initial brain influx rate and reaching mainly the brain parenchyma (95%) after their penetration, with negligible efflux after 15 min of intra-cerebroventricular injection. Therefore, BEA and ENs are able to reach systemic circulation through various routes of exposure and may exert central nervous system (CNS) effects passing the blood-brain barrier (BBB) [115].

#### 2.7.5. NIV and FUS-X

NIV was poorly absorbed orally with low bioavailability (4%) and rapidly eliminated via feces in chicken after oral administration (0.8 mg/kg bw). Elimination half-life was 2.51 h and 5.27 h and after oral and IV administration, respectively. NIV was detected in small intestine, kidney, heart, liver, and muscle suggesting that it is absorbed from the gastrointestinal tract diffusing into various broiler tissues [125]. FUS-X and NIV were detected in pig plasma, urine, feces, and tissues after a single IV and oral administration (1 mg/kg bw), and in vital organs (24 h after oral administration), with highest FUS-X concentration in liver (166 ng/g), kidney, (66.3 ng/g), and spleen (7.4 ng/g) 3 h after oral administration [124].

#### 2.7.6. T-2 and HT-2

T-2 toxin was rapidly absorbed and metabolized into HT-2 and T-2 triol after oral and IV administration (1 mg/kg bw) in pig. HT-2 and T-2 triol metabolites were rapidly distributed into tissues, mainly liver (216.3 µg /kg), kidney (206 µg /kg), and the small intestines (140.5 µg /kg), still detected at 6 h after administration. The highest T-2 concentration were detected in fat tissues (58.6 µg g/kg), lungs (54.0 µg/kg), and spleen (47.8 µg /kg). T-2 was quickly eliminated in plasma after IV administration, and low urine excretion (<7%) was shown for T-2, HT-2, and T-2 triol, with only HT-2 (0.25%) being detected in feces [126]. T-2 volumes of distribution, total body clearance and elimination half-life was 0.14 L/kg, 0.03 L/min kg, and 31.8 min after oral administration (0.02 mg/kg bw) in broiler chickens [113]. Trace concentrations of T-2, NEO and T-2-triol, as well as large amount of HT-2 were detected in chicken muscle and liver after oral administration (3 mg/kg of bw) indicating that T-2 toxin was rapidly metabolized to mainly HT-2 as the main metabolite, which was even detected in liver 48h after administration [129]. Conversely, non-residual T-2 toxin was neither detected in any rat organ nor tissues even at high exposure level (20 mg/kg) in rat [123].

#### 2.7.7. FBs

FB1 bioavailability was 3.2% after single-dose oral administration (100 mg/kg bw), with elimination half-life, mean residence time, and clearance of 214 min, 408 min, and 7.5 mL/min/kg after oral bolus, respectively, and 85 and 52 min, and 7.5 mL/min/kg after IV administration (10 mg/kg bw). Liver and kidney contained the highest levels of FB1 after 24 h IV (liver: 46, kidney: 50 µg/kg) and oral administration (liver: 5458, kidney: 5785 µg/kg), being non detectable in muscle. Persistence of FB1 was observed after 9 weeks of FB1/FB2 feed supplementation (5–20 mg/kg) showing liver (11,922 µg/kg) and kidney (22 µg/kg) residues 8 h after the last intake [138].

### 2.8. Risk Assessment

Since the consumption of contaminated food is considered the major source of human mycotoxin exposure, accurate estimation of mycotoxin exposure is compulsory to facilitate weighty risk assessment. The measurement of specific urinary mycotoxin biomarkers—both the metabolite generated by human metabolism or the parent toxin itself—is a valid alternative to measure mycotoxin exposure whenever biomarkers excretion correlates well with mycotoxin intake [77]. Thus, mycotoxin exposure assessment throughout biomonitoring studies based on mycotoxin analysis in human biological samples such as urine, serum and breast milk, have provided useful information over recent years, OTA, DON, and CIT being the most reported mycotoxins.

#### 2.8.1. OTA

Analyzing swine serum samples was presented as an alternative approach to monitor the presence of OTA in feed to prevent the occurrence of ochratoxicosis in animal production. A direct relationship between OTA exposure levels and serum concentrations in slaughtered swine (*n* = 400) was reported in Brazil [40]. OTA exposure was also estimated based on human serum OTA levels carried out in several studies worldwide. OTA and OT-α were detected in 100% and 95% of the analyzed plasma samples in Bangladesh (*n* = 64) at ranges of 0.20–6.63 µg/L, and 0.10–0.79 µg/L, respectively. The calculated OTA intake on the basis of plasma concentration in the population was lower than the tolerable weekly OTA intake (120 ng/kg b.w/wk) set by EFSA [143]. Moreover, non-significant association was observed between OTA serum levels with the intake of typical staple foods in Bangladesh [58]. OTA was detected in 77% of the analyzed serum samples in Chile (*n* = 88) at concentrations lower than 1 µg/L. The OTA continuous dietary intake was in all cases lower than the TDI defined by the International Scientific Committee on Ochratoxin A in 2002 [144]. Correlations between OTA levels in plasma and food consumption were not significant [44]. One hundred percent of OTA frequency was reported in serum samples from Spain (*n* = 168) in a range of 0.15–5.71 µg/L. OTA intake did not exceed the tolerable weekly OTA intake. Non-correlation was observed between the OTA serum levels and the individual consumption of 26 food groups described as possibly contaminated with OTA in the Spanish population [36]. In the German cohort OTA and OTα were detected in 100% and 78% of all analyzed urines (*n* = 50) ranging between 0.02–1.82 µg/L, and 0.01–14.25 µg/L, respectively, indicating that the unmetabolized OTA excretion in urine represents only a small fraction (<3%) of the ingested dose [62]. OTA was detectable in 80% and 50% of infant urine samples from Germany (*n* = 10) and Turkey (*n* = 28) with concentrations ranging from 30 to 1360 ng/L confirming its frequent exposure in this group of under 2 year olds [64]. In Czech Republic OTA was found in almost all analyzed serum samples from pregnant women (*n* = 115) in concentrations up to 1.13 µg/L [43], and in women of the child rearing age (*n* = 100) up to 0.35 µg/L [38]. Czech Republic data from serum correlate with OTA dietary exposure assessment. OTA levels in pregnant women serum did not show significant difference from normal population data. OTA plasma levels detected in an assessment study carried out on German grain workers (*n* = 61) ranged between 0.07 and 0.75 µg/L. Evidence of a significant inhalatory burden of OTA was not found in grain workers [35]. OTA was investigated in the etiology of bladder cancer in Pakistan patients (*n* = 87) and healthy individuals (*n* = 30). Ninety two percent of the analyzed serum samples showed concentrations up to 3.4 µg/mL and 1.2 µg/mL, respectively, and non-association was evidenced [37]. Data found in Tunisia seemed to relate chronic interstitial nephropathy and OTA. Food and serum OTA levels were significantly different from the healthy and nephropathy groups [41].

The analysis of OTA in urine is an appropriate biomarker and a very useful tool to monitor OTA exposure of populations. As urine collection is less invasive than blood, urinary studies for population exposure assessment have been widely performed. Ninety three percent of the analyzed pregnant women urine samples from Bangladesh (*n* = 54) were positive for OTA in concentrations lower than 0.84 µg/L, similar levels to those determined recently in the general population of this country [96]. OTA was found in 42 (*n* = 60) and 27 (*n* = 120) urine samples analyzed in Portugal at concentrations lower than 0.105 µg/L [60] and 0.208 µg/L [61], respectively, whereas 51 (*n* = 122) urine samples from Spain were positive at concentrations lower than and 0.124 µg/L. OTA urinary ranges in both populations were comparable to those found in other countries in Europe such as Italy and UK. However, for most countries a great variation in the range of OTA levels was observed [61]. OTA was present in 35% (*n* = 239) and 70% (*n* = 32) of adult urine samples from Belgium at lower concentrations (pg/mL). Estimated OTA PDI exceeded the TDI for OTA in 1% of the studied Belgium population [74,105].

Detecting OTA levels in breast milk may provide valuable information about the exposure degree of both mother and baby, and it is useful for the estimation of overall risk characterization. In this way, breast milk samples from Turkey (*n* = 75) were analyzed indicating a high exposure level of mothers to OTA. One hundred percent of samples showed contamination in the range of 0.6–13.1 µg/L, representing a potential hazard of OTA to infants as well as their mothers [106].

#### 2.8.2. DON and Metabolites

DON and its metabolites were detected in 58 analyzed urine samples from China (*n* = 60) in concentrations up to 30.5 µg/g creatinine. Urinary DON was not significantly associated with rice intake [84]. DON was reported in 52% of pregnant women urine samples from Bangladesh (*n* = 54) in levels ranging 0.18–7.16 µg/L. No individual had an estimated daily DON intake above the provisional maximum tolerable daily intake (PMTDI) of 1 µg/kg b.w. set by the WHO/JECFA (2011). Moreover, DON exposure in pregnant women in Bangladesh appears to be less and lower than observed in biomonitoring studies performed in Europe and Africa [85]. On the other hand 58% of Bangladeshi adult urine samples (*n* = 164) were positive for DON in the rage of 0.16–1.78 µg/L, while German analyzed urine samples (*n* = 50) contained DON (100%) and DOM-1 (40%) in concentrations up to 38.44 µg/L and 0.73 µg/L, respectively [86]. The mean DON intake in individuals from both Bangladesh and Germany was lower than the PMTDI. However, the mean DON level in German urine samples was about 53-fold higher than that found in Bangladeshi samples indicating a low and high dietary DON exposure among the adult population in Bangladesh and Germany, respectively. Moreover, DON (29%) and DON-3-GlcA (82%) were detected in the analyzed urine samples from Germany (*n* = 101) at concentrations up to 31 and 139 mg/g creatinine, respectively. The mean DON PDI of 12% samples exceeded the established value [68]. DON (22%) and DON-GlcAs (96%) were detected in urine samples from Austria (*n* = 27) with an average concentration (DON + DON-GlcAs) of 20.4 µg/L. Thirty three percent of the individuals exceeded the DON PMTDI value according to their DON urinary levels [72]. Thirty seven urine samples from Spain (*n* = 54) showed DON concentrations up to 69.1 µg/g creatinine. Based on DON urinary levels 8.1% of the volunteers from Spanish volunteers, as well as, 2 out of 9 exposed children, were estimated to exceed the DON PMTDI [93]. The overall DON incidence in Belgium urine samples was 70% for children (*n* = 155) and 37% for adults (*n* = 239) in concentrations up to 27 and 327 ng/mg creatinine, respectively. The calculated DON PDI possibly exceeded the PMTDI in 16–69% of the population [74]. All analyzed urine samples (*n* = 32) from Belgium contained concentrations of DON (60%) at ng/L or its metabolites DON-15-GlcA (100%), DON-3-GlcA (90%) and DOM-1-GlcA (25%) This emphasizes the importance of glucuronidation for detoxification of DON in humans [105].

#### 2.8.3. CIT

CIT and HO-CIT were detected in 94% and 71% of the analyzed urine samples from Bangladesh (*n* = 69) in concentrations up to 1.22 and 7.47 µg/L, with significantly higher levels in the rural cohort compared to the urban cohort. However, it is unclear, whether this biomarker result reflects a difference in food habits and/or an additional occupational exposure [88]. Eighty seven percent of the analyzed pregnant women urines from Bangladesh (*n* = 54) were positive for CIT in concentrations lower than 6.93 µg/L. Based on urinary concentrations the calculated CIT PDI of 9% of the Bangladeshi pregnant women exceeded the preliminary tolerable value set by the European Food Safety Authority (0.2 μg/kg/day) [96,145]. In the German population 82% and 84% of the analyzed urine samples (*n* = 50) contained these mycotoxins with maximum concentrations of 0.1 and 0.5 µg/L indicating a widespread and variable CIT exposure [87]. With regard to Belgium, CIT and/or OH-CIT were detected in lower concentrations (pg/mL) in 90% of the analyzed urine samples (*n* = 32) indicating that humans are much more exposed to CIT than was realized before [105]. Moreover CIT was present in 72% and 59% of Belgium urine samples from adults (*n* = 239) and children (*n* = 155), respectively, with low average concentrations (<73.3 pg/mg creatinine). Despite the mean detected concentration of HO-CIT being tenfold higher than CIT, a lower prevalence (6% and 12% for children and adults, respectively) was reported [74].

#### 2.8.4. Multi-Mycotoxins

Apart from these most studied compounds, other relevant mycotoxins have been the focus of several biomonitoring studies, including AFs, FBs, ZON, and ENs. Eight mycotoxins including AFM1, DH-CIT, DON, DON-GLcA, EN B, FB1, OTA, and α-ZEL were detected in urine samples from Bangladesh (*n* = 95), Germany (*n* = 50), and Haiti (*n* = 142). DON and DON-GlcA were exclusively detected in urines from Germany and Haiti whereas urinary OTA and DH-CIT concentrations were significantly higher in Bangladeshi samples. AFM1 was present in samples from Bangladesh and Haiti only. The mean PDI was below the TDI for FB1, AFB1, and ZON, however calculated DON PDI exceeded the PMTDI in 6% of the samples from Germany (2/50) and Haiti (4/142) [69]. Seventy three percent of the analyzed children urine samples from Cameroon (*n* = 220) were positive for OTA (32%), DON (17%), AFM1 (14%), FB1 (11%), β-ZOL (8%), ZEN (4%), α-ZOL (4%), and DON-3-GlcA (1%) in concentrations up to 77 µg/L, indicating that children in Cameroon under the age of five are exposed to high levels of carcinogenic substances such as FB1, AFM1, and OTA through breastfeeding [141]. A total of eight mycotoxins were detected in 51% of the analyzed urine samples from Nigeria (*n* = 120), with OTA (28.3%), AFM1 (14.2%), and FB1 (13.3%) being the most frequent ones. The mean estimated OTA daily intake (0.01 μg/kg bw/day) in the Nigerian population was close to the suggested TDI of 0.017 μg/kg bw/day derived from the tolerable weekly intake recommended by EFSA (2006). The estimated mean AFB1 intake was 0.67 μg/kg bw/day (max = 2.5 μg/kg bw/day), whereas the mean estimated FB1 intake was 35 μg/kg bw/day (max = 76 μg/kg bw/day), a level significantly greater than the recommended TDI of 2 μg/kg bw/day [70,146]. Non detectable levels of FB1 and FB2 were observed in the analyzed human urine samples obtained from Portugal (*n* = 68) [89]. The presence of ZON + ZOLs (100%), OTA (100%), DON (96%), FB1 (56%), and AFM1 (6%) were reported in urine samples from Italy (*n* = 52) in concentrations up to 67 µg/L. The estimated human exposure to FB1 and ZON was largely below the TDI, however 94% and 40% of the Italian volunteers exceeded the TDI for OTA and DON, respectively [77]. The duration of AFB1 exposure in bakers from Egypt (*n* = 290) was significantly correlated with serum concentrations [50].

All analyzed breast milk samples from Italy (*n* = 47) were positive for ZON (0.26–1.78 µg/L) [111]. However, from the analyzed breast milk samples from Iran (*n* = 136) only one sample showed contamination with AFM1 and two with OTA at low concentrations (ng/L) [107]. Only two breast milk samples from Brazil (*n* = 224) were positive for AFB2 (0.005 µg/L), indicating non-infant risk derived from AFs and OTA exposure [109]. Although mycotoxins may be transferred from maternal blood to milk, breast milk is comparably rarely evaluated even though the limits of mycotoxins in infant food are highly restrictive and controlled by monitoring programs. Warth et al. [147] reviewed the current situation of mycotoxins in human breast milk reporting studies mainly focused on AFs and OTA in different locations such as Iran, Turkey, Egypt, Chile, Nigeria, Brazil, Tanzania, Cameroon, Germany, Italy, Poland, etc. However, little is still known about the pattern of mycotoxins and their metabolites in breast milk as well as lactational transfer rates or potential combinatory effects.

#### 2.8.5. Mycotoxin Binders

The prevention of fungal infections is the most rational and efficient way to avoid mycotoxins in agricultural commodities, however, under certain environmental conditions mycotoxin contamination is unavoidable. Several studies have shown adsorbent materials (mycotoxin binders) with large affinity for mycotoxins by the formation of stable linkages, but most of them seem to only bind a small group of mycotoxins while demonstrating very little or no binding to others [148]. Some mycotoxin bindings can efficiently adsorb mycotoxins and they have already shown their efficacy in in vivo studies. For instance, activated carbon (1 g/kg bw) significantly reduced the absorption and oral availability of DON after oral bolus (0.750 mg DON/kg bw) in chicken [46] and even lower doses (0.1 g/kg bw) completely prevented DON absorption in pig after single oral bolus (0.05 mg/kg bw) [54]. Conversely, glucomannan mycotoxin adsorbent (2 kg/ton diet) did not prevent DON absorption (no significant differences of DON and DOM-1 plasma concentrations) at the dietary inclusion level (4–6.5 mg/kg) in turkey poults [47] and commercial mycotoxin adsorbent lack of protective effect against STE adsorption in cattle after 72 days STE diet supplementation (0.01–0.24 mg/kg) [65]. In this way, the use of mycotoxin binders may be valuable when other preventive measures against molds and mycotoxins have failed. However, the selection of the appropriate adsorbent substance must be done considering its mycotoxin adsorption efficacy or mold inhibition, the safety to animals and humans, having high stability and ability to face diverse conditions during feed/food mixing, as well as cost effectiveness [149].

## 3. Conclusions

The latest studies of mycotoxin determination in biological samples—fluids, tissues and organs—were collected, studied, and summarized. Considering the great majority of biological samples the most common extraction technique used for mycotoxins extraction was LLE in a single step or followed by a SPE clean-up. Nevertheless, reported mycotoxin analyses in urine were mainly based on dilute-and-shoot, IAC and combinations of SPE-IAC and LLE-SPE techniques. Acetonitrile, ethyl acetate, dichlorometane, and methanol were the most common organic solvents employed for mycotoxin extraction. With regard to detection techniques, LC-MS/MS systems were the most commonly used—among other alternatives such as LC-FD, GC-MS/MS, and ELISA—especially in the case of urine samples where a higher number of compounds was simultaneously determined. The species from which the biological samples were collected were mainly human (non-invasive collection samples), followed by pig, rat, and chicken. The most analyzed mycotoxin was OTA, followed by DON-ZON and their metabolites, AFs, FBs, ENs-BEA, T-2, and HT-2. The most common studies of mycotoxin analysis in biological samples were focused on method development and human biomonitoring, followed by toxicokinetics, absorption, metabolism, and bioavailability studies. New insights of mycotoxin bioavailability, toxicokinetics, ADME, bioaccumulation, and tissue persistence have been obtained through the analysis of biological samples, mainly focused on DON, ZON, OTA, ENs and BEA, T-2, NIV, and FB1. Despite the relatively high frequency of mycotoxins detected in biological samples from biomonitoring studies data, calculated PDIs were generally below the established TDIs. However, 24% of the studies reported TDIs above the established values in a variable percentage of individuals, with DON as the most representative. DON PDI raised PMTDI in regions such as Haiti, Germany, Spain, Austria, Italy, and Belgium. Also the children population showed DON PDI exceeding the established level in Spain and Belgium. On the other hand, OTA PDI exceeded TDI in Belgian adults and children, and in Italian adults. Finally, CIT PDI was higher than TDI for pregnant women from Bangladesh, while TDI for FB1 was exceeded in some of the individuals from Nigeria. Finally, it is worth noting that the future in mycotoxin detection from biological samples seems to be pointing towards aptasensors because of their specificity, sensitivity, and easy use. The difficulty of biomonitoring studies—samples of reduced volume or size with very low concentrations of mycotoxins—may be solved with this newfangled experimental approach. These results evidence the importance of biological sample analysis as a useful tool for human and animal exposure assessment to mycotoxins.

## 4. Materials and Methods

### Data Resource

An outspread search strategy was follow to obtaining abstracts in the databases Web of Science (WOS), PUBMED, and MedLine with the last databases access on July 2017. Abstracts were analyzed to include only study types with relevant information and the selected studies were identified. The search was performed using the following keywords and topics to find literature of interest: mycotoxin analysis in biological samples, biological fluids, serum, urine, feces, organs, tissues; mycotoxin carry over; mycotoxin bioaccumulation; mycotoxin biomarkers, biomonitoring, transference, ADME, etc.

The search was framed between the years 2005 and 2017 to focus in recent literature and refine the study in the last decade. From the identified publications, relevant data was found in 114 articles. To facilitate data presentation, the studies were classified based on the analyzed biological sample(s). Thus, four groups were established: (i) serum; (ii) urine; (iii) minor biological fluids and fluids combinations (including feces); (iv) organs and tissues. The information was checked to select bibliographies of relevant literature, and a thorough evaluation was performed to summarize information about extraction methodology, detection techniques, sample size, limits of detection-quantitation, studied mycotoxins, main purpose(s) of the biological sample analysis, and animal species of biological sample origin.

## Figures and Tables

**Table 1:** Studies of one single mycotoxin and multi-mycotoxin determination in serum.

Species	Volume (μL)	Mycotoxin	Extraction Procedure	Detection Technique	LOD (μg/L)	LOQ (μg/L)	Reference
*Single mycotoxin*							
Human	50	OTA	LLE: CH_2_Cl_2_	ELISA vs. CE-LIF (CE/laser-induced FD)	0.5	-	[39]
Human	-	OTA	IAC	HPLC-FD	-	-	[42]
Human	-	OTA	SPE: Sep-Pak RP-18. IAC: Ochraprep	HPLC-FD	0.1	0.4	[44]
Human	-	OTA	LLE: CHCl_3_/HCl	HPLC-FD	0.05	-	[35]
Human	2000	OTA	LLE: CHCl_3_	HPLC-FD LC-ESI-MS/MS	0.01	0.07	[36]
Human	6000	OTA	SPE: C18	HPLC-FD	0.1	0.2	[41]
Human	1000	OTA	LLE: CHCl_3_, SPE	HPLC-FD	0.05	-	[37]
Human	2000–3000	OTA	LLE: CHCl_3_, IAC: Ochraprep	ELISA and HPLC-FD	-	0.050	[38]
Human	-	OTA	IAC: Ochraprep	HPLC-FD	-	0.1	[43]
Human	-	AFB1	IAC: Easi-Extract Aflatoxin	ELISA	-	-	[50]
Rat	100	ZON	LLE: TBME	LC-MS/MS	-	0.5	[48]
Rat	100	ZON	LLE: TBME	HPLC-FD	-	10	[49]
Swine	800	OTA	LLE: CH_2_Cl_2_	HPLC	0.1	-	[40]
Chicken	250	DON	LLE: ACN	LC-MS/MS	0.1–0.2	1	[46,47]
Horse	2000	OTA	LLE: CH_2_Cl_2_, IAC: Ochratest	ELISA, HPLC-FD	0.015	-	[21]
*Multi-mycotoxin*							
Human	500	OTA, OTα	LLE: CHCl_3_/isopropanol	HPLC-FD	0.05	0.1	[58]
Chicken	20–250	EN B, EN B1	LLE: ACN	LC/MS/MS, UHPLC-HRMS	0.000091–0.00017	0.025	[45]
Pig	250	EN A, A1, B, B1	LLE: ACN	LC-MS/MS	0.01–0.001	0.1–0.2	[52,53]
Pig	250	DON, T-2, HT-2, OTA, FB1, AFB1, ZON, ZAN, α-ZOL, β-ZOL, α-ZAL, β-ZAL, DOM-1	LLE: ACN	LC-MS/MS	0.01–0.52	0.5–10	[51,54]
Chicken, pig, laying hens, turkey poults	250	ZON, α-ZOL, β-ZOL, ZAN, α-ZAL, β-ZAL	LLE: ACN	LC–MS/MS (U)HPLC–HR-MS	0.004–0.07	0.2–5	[55,57]
Chicken, Pig	250	DON, 3-ADON, 15-ADON, DOM-1	LLE: ACN	LC–MS/MS	0.01–0.7	0.1–2	[56]
Fish	250	AFB1, AFB2, AFG1, AFG2, OTA, FUS-X, STG, FB1, FB2, FB3, BEA, EN A, EN A1, EN B, EN B1	DLLME: ACN/EtOAc	LC/MS/MS	0.1–12.0	1.5–17.0	[59]

**Table 2:** Studies of one single mycotoxin and structurally related mycotoxins determination in urine.

Species	Sample Volume	Mycotoxins	Extraction Procedure	Detection Technique	LOD (μg/L)	LOQ (μg/L)	Reference
*Single mycotoxin*							
Human	10 mL	OTA	IAC: OchraTest	HPLC-FD	-	0.02	[60]
Human	1 mL (0.5 mL)	OTA	SPME	HPLC-ESI-MS/MS	0.3	0.7	[63]
Human	10 mL	OTA	IAC: OchraTest	HPLC-FD	-	0.007	[61]
Human	5 mL	OTA, OTα	IAC	HPLC-FD	0.05	0.1	[62]
Human	20 mL	AFB1-N7-Gua	SPE: MCX, IAC: Bond elute LRC, SPE: C18	HPLC-ESI-MS/MS	-	-	[67]
Human	0.5 mL	DON-GlcA	Dilute-shoot: ACN/H_2_O	LC-MS/MS	3–6	10–20	[66]
Cattle	0.5 mL	STG	SPE: C18	LC-MS/MS	-	-	[65]
*Multi-mycotoxin*							
Human	1 mL	DON, DOM-1	IAC: Wide Bore DON	LC-MS/MS	0.5	-	[84]
Human	0.1 mL	DON, DON-GlcAs	Dilute-shoot: ACN/H_2_O	LC-MS/MS	4–10	13–33	[72]
Human Rat	0.4	DON, DOM-1, DOM-1-G, DON-G1, DON-G2	SPE: Strata-X	HPLC-APCI-MS/MS	1–2	3–6	[76]
Human	10 mL	FB1, FB2	PBS, IAC	HPLC-ESI-MS/MS	5	10	[89]
Human Swine Bovine	10 mL	ZON, α-ZOL, β-ZOL, ZAN, α-ZAL, β-ZAL	SPE: C18	HPLC-EC	1.3–1.4	4.2–4.8	[75]
Bovine, swine	5 mL	ZON, α-ZOL, β-ZOL, ZAN, α-ZAL, β-ZAL	IAC	HPLC-ESI-MS/MS	0.56–0.68 (CCa)	-	[90]
Bovine	5 mL	ZON, α-ZOL, β-ZOL, ZAN	LLE: TBME, Hx, SPE: C18, SPE: -NH2, derivatization	GC-MS	CCa: 0.06–0.35	CCb: 0.11–0.60	[78]
Human	5 mL	CIT, HO-CIT	IAC	LC-MS/MS	0.02–0.05	0.05–0.1	[87,88]
Human	5 mL	DON, DOM-1	IAC	LC-MS/MS	0.10–0.16	0.2–0.3	[85,86]
Human	5 mL	OTA, OTA-GlcA, OTA-sulfates	LLE: CHCl_3_-Isopropanol	LC-MS/MS	0.1–0.5	0.5–1	[64]

CCa: decision limit; CCb: detection capability.

**Table 3:** Studies of multi-mycotoxin determination in urine.

Species	Volume (mL)	Mycotoxins	Extraction Procedure	Detection Technique	LOD (μg/L)	LOQ (μg/L)	Reference
Human	10	AFM1, FB1, FB2, OTA, OTα	IAC	HPLC-ESI-MS/MS	0.001–0.045	0.004–0.135	[82]
Human	10	AFB1, AFB2, AFG1, AFG2, OTA, DON, ZON, FB1, FB2, T-2, HT-2	IAC	LC-QTRAP-MS/MS	0.4–10	1.2–35	[83]
Human Pig	6	AFM1, OTA, DON, DOM-1, α-ZOL, β-ZOL, FB1	SPE: Oasis HLB, IAC	HPLC-Qtrap-MS/MS	0.001–2.2	0.002–4.4	[80]
Human Pig	5	DON, NEO, AFB1, AFM1, HT-2, HT2, OTA, OTα, ZON, α-ZOL, β-ZOL, FB1	SALLE: MgSO_4_, EtOAc-ACN	LC-MS/MS	0.01–0.5	0.07–3.3	[30]
Pig	6	DON, DOM-1, OTA, AFB1, AFM1, FB1, ZON and α-ZOL	Myco6in1 IAC-Oasis HLB SPE	LC-MS/MS	-	-	[81]
Human	10	DON, OTA, FB1, AFB1, ZON, T-2, HT-2, AFB1, CIT, DOM, DON-2-GlcA, ZON-14-GlcA, α-ZOL, β-ZOL, 4-OH-OTA, OTα, AFM1, AFB1-N7-Gua	LLE: EtOaC/FA, SPE: SAX	LC-MS/MS	0.01–3.65	0.02–5.76	[79]
Human	0.1	DON, DON-3-GlcA, DON-15-GlcA, DOM-1, NIV, T-2, HT-2, ZON, ZON-14-O-GlcA, α-ZOL, b-ZOL, FB1, FB2, OTA, AFM1	Dilute-shoot: ACN/H_2_O	HPLC-ESI-MS/MS	0.005–2	0.017–6.7	[71]
Human	-	DON, DON-3-GlcA, DON-15-GlcA, ZEN, ZEN-14-GlcA.	Dilute-shoot: ACN/H_2_O	LC-MS/MS	0.2–4	0.3–6	[104]
Human	6	DON, DOM-1, AFM1, FB1, ZON, α-ZOL, β-ZOL, OTA	SPE: Myco6in1^®^ and OASIS^®^ HLB columns	UPLC-MS/MS LC-QTrap MS/MS UPLC-API 5000 MS/MS	-	0.02–4.4 0.006–9.9	[77]
Human	0.1	AFM1, OTA, FB1, DON, DON-GlcAs, FB2, DOM-1, ZON, ZON-14-GlcA, α-ZOL, β-ZOL, T-2, HT-2, NIV	Dilute-shoot: ACN/H_2_O	LC-MS/MS	0.05–12	0.15–40	[70]
Human	10	*LLE*, *SPE:* AFB1, AFB2, AFG1, AFG2, AFB1-N7-gua, AFM1, CIT, DON, DON-3-GlcA, DOM-1, FB1, HFB1, OTA, OTα, 4-OH-OTA, T-2, HT-2, ZON, ZON-14-GlcA, α-ZOL, β-ZOL. *Filter-shoot:* AFB1, AFB2, AFG1, AFG2, AFM1, CIT, OH-CIT, DON, DON-3-GlcA , DON-15-GlcA, DOM-1, DOM-1-3-GlcA, 3-ADON, 3-ADON-15-GlcA, 15-ADON, 15-ADON-3-GlcA, DAS, FB1, FB2, FB3, FUS-X, OTA, OTα, T-2, HT-2, ZON, ZON-14-GlcA, α-ZOL, α-ZOL-7-GlcA, α-ZOL-14-GlcA, β-ZOL, β-ZOL-14-GlcA.	LLE: EtOAc/FA, SPE Filter-shoot	LC-MS/MS	-	-	[73,74]
Human	10	AFB1, DAS, FusX, 3-AcDON, 15-AcDON, β-ZEL, α-ZEL, CIT, OTα, DOM-1, FB1, FB2, FB3, DON, ZEN, T2, HT2, DON-3-GlcA, DOM-GlcA, ZEN-14-GlcA, β-ZEL-7-GlcA, β-ZEL-14- GlcA, α-ZEL-7-GlcA, α-ZEL-14-GlcA, 15-AcDON-3-GlcA, 3-AcDON-15-GlcA, OTA, CIT and AFM1	Filter-shot IAC (OTA, CIT, AFM1)	LC-MS/MS	0.001–0.2	0.003–0.5	[105]
Human	0.1	DON, DON-3-GlcA, T-2, HT-2, HT-2-4-GlcA, FB1, AFB1, AFB2, AFG1, AFG2, AFM1, ZON, ZAN, α-ZOL, β-ZOL, ZON-14-GlcA, ZAN-14-GlcA, α-ZOL-14-GlcA, β-ZOL-14-GlcA, OTA, OTα, EN B, DH-CIT	Dilute-shoot: H_2_O/ACN/FA	LC-MS/MS	0.0005–0.3125	0.0013–0.3125	[68]
Human	0.1	DON, DON-3-GlcA, T-2, HT-2, HT-2-4-GlcA, FB1, AFB1, AFB2, AFG1, AFG2, AFM1, ZON, ZAN, α-ZOL, β-ZOL, ZON-14-GlcA, ZAN-14-GlcA, α-ZOL-14-GlcA, β-ZOL-14-GlcA, OTA, OTα, EN B, DH-CIT	Dilute-shoot: H_2_O/ACN/FA	LC-MS/MS	0.000125–0.45	0.0005–0.9	[69]
Human	10	DOM-1, DON, 3-ADON, FUS-X, DAS, NIV, NEO, HT-2, T-2, ZON, α-ZOL, β-ZOL, ZAN, α-ZAL, β-ZAL	QuECHERS, d-SPE	GC-MS/MS	0.12–4	0.25–8	[31,92,93]
Human	1	DON, DOM-1, 3-ADON, 15-ADON, ZON, α-ZOL, β-ZOL, ZAN, α-ZAL, β-ZAL	SALLE: ACN, NaCl-C18	GC-MS/MS	0.12–4	0.25–8	[94]

**Table 4:** Studies of multi-mycotoxin determination in minor biological fluids and fluid combination.

Species	Sample	Volume	Mycotoxins	Extraction Procedure	Detection Technique	LOD (μg/L)	LOQ (μg/L)	Reference
Human	Breast milk	1 mL	OTA	LLE: CHCl_3_	HPLC-FLD	0.01	-	[106]
Human	Breast milk	1 mL	AFM1, OTA	LLE: CHCl_3_, ACN	ELISA, HPLC-FD	-	-	[107]
Human	Breast milk	5 mL	ZON	IAC	ELISA HPLC-FLD	0.06 0.02–0.05	-	[111]
Human	Breast milk	10 mL	DON, 3-ADON, NIV, FUSX, NEO, DAS, HT-2, T-2, ZON, α-ZOL, β-ZOL, FB1, FB2, FB3, EN A, EN A1, EN B, EN B1, BEA, AFB1, AFB2, AFG1, AFG2, AFM1, STG, OTA, OTα	QuEChERS	UHPLC-HRMS	-	1–50	[110]
Cow	Milk							[24]
Donkey	Milk		AFM1 and AFM2	IAC	HPLC-FLD, LC-MS/MS			[23]
Cow	Milk	50 mL	AFM1	IAC	LC-MS/MS	-	0.01	[22]
Human	Serum Urine	1 mL 5mL	OTA, OTα	LLE: CHCl_3_/isopropanol	HPLC-FLD HPLC-ESI-MS/MS	0.07 0.02	0.1 0.5	[95,96]
Human	Serum Urine	1 mL 5 mL	CIT	LLE: ACN, IAC: CitriTest	HPLC-FLD			[96,97,98,99]
Human	Serum Urine	250 μL 5 mL	ENs, BEA	LLE: MeOH/H_2_O, SPE: GCB	LC-MS/MS	0.01–0.0025–0.02	0.02–0.04 0.005–0.02	[98]
Chicken Pig	Serum Bile	250 μL 1 mL	DON, DOM-1, T-2, HT-2	LLE: MeOH, SPE LLE: MeOH/H_2_O, EtOAc	LC-MS/MS	0.01–0.63	1.0–2.5	[112,113]
Pig	Serum, urine, liquor	500 μL	ZON, DON, ZAN, α-ZOL, β-ZOL,α-ZAL, β-ZAL	SPE: Oasis HLB	HPLC-ESI-MS/MS	0.005–0.71 0.03–0.16 0.02–0.21	0.08–2.37 0.1–0.52 0.07–0.70	[97]
Rat	Serum, urine, feces	200 μL 100 mg	EN A	LLE: EtOAc	LC-MS/MS	1.8–2.3	5.4–7	[101]
Rat	Serum, urine, feces	500 μL 500 mg	EN A, A1, B, B1	LLE: ACN	LC-MS/MS	0.2–1	2–10	[100]
Horse	Serum, urine, feces	1 mL 5 mL 2 g	ZON, DON, ZAN, α-ZOL, β-ZOL, α-ZAL, β-ZAL	IAC SPE: C18 , IAC SPE: C18 , IAC	HPLC-APCI-MS/MS	0.1–0.3 0.1–0.2 0.1–0.5	0.5–0.6 0.5–1 0.5–1	[102]
Human	Feces	1–2 g 1 mL	OTA, OTB	SPE: C18	HPLC-FLD	-	1.25–2.22 1.44–2.99	[108]
Human	Breast milk	2 mL	AFB1, AFB2, AFG1, AFG2, AFM1, OTA	LLE: ACN/EtOAc, LTP (low temperature purification)	HPLC-FLD LC-MS/MS	-	0.005–0.03	[109]

**Table 5:** Studies of single mycotoxin determination in organs and tissues.

Species	Biological Sample	Sample Size	Mycotoxins	Extraction Procedure	Detection Technique	LOD (μg/kg)	LOQ (μg/kg)	Reference
Mouse	Plasma, spleen, liver, lung, and kidney	40–200 mg	DON	PBS	ELISA	-	-	[19]
Mouse	Plasma, liver, kidney, heart, spleen, and brain	100 μL (extract)	DON	ice-cold ethanol/trichloroacetic acid	ELISA	-	-	[139]
Rat	Plasma, liver, kidney	250 μL 200–400 mg	OTA	LLE: ACN, SPE	HPLC-FLD	1–14.3	8.4–52.8	[121]
Pig	Plasma, liver, kidney	800 μL 20 g	OTA	LLE: ACN, SPE	HPLC-FD and LC-MS/MS	0.14 0.25	0.25 0.5	[120]
Swine, cattle, sheep, horse, fish, chicken, turkey, geese, duck	Muscles, liver and kidneys	10 g	OTA	LLE: CHCl_3_, IAC: OchraTest	LC-FD	-	0.2	[134]
Laying Hens	Kidneys, liver, bile (eggs)	2.5 g 200 μL	OTA	LLE: CHCl_3_, SPE	HPLC-FD	0.3–0.5	1	[27]
Turkey poults	Plasma, muscle, liver, and kidney	250 μL 1 g	FB1	SPE: SAX IAC: FumoniPrep	HPLC-FLD	13	25	[138]
Rat	Serum, bile, and urine. Lung, liver, spleen, kidneys, heart, testes, brain, muscle, adipose tissue, stomach, and small intestine		ZAN	LLE, IAC	LC-MS/MS HPLC-FD	-	0.5 10	[122]
Rat	Liver and kidney	1 g	T-2	LLE: ACN, SPE: charcoal/celite/aluminium trioxide	HPTLC	-	100	[123]
Rat	Serum, stomach, duodenum, jejunum, ileum, colon, liver	0.5 g 0.5 mL	EN A	LLE: EtOAc	LC-MS/MS	200	600	[118]
Chicken	Serum, liver, kidney, heart, muscle, small intestine, and excreta	1 mL 5 g	NIV	LLE: ACN/H_2_O (NH_4_)_2_SO_4_, SPE C18	LC-MS/MS	-	2–2.5	[125]
Cow	Serum, milk, liver, kidney, muscles, fat, and jejuno, ileum	2–5 mL 10 g	OTA	LLE: ACN/Hex, IAC	HPLC-FD	-	-	[133]

**Table 6:** Studies of structurally related mycotoxins determination in organs and tissues.

Species	Biological Sample	Sample Size	Mycotoxins	Extraction Procedure	Detection Technique	LOD (μg/kg)	LOQ (μg/kg)	Reference
Swine	Plasma, liver	10 g	FB1, AP-1	LLE: ACN, SPE: C18, SAX, Oasis HLB. LLE: ACN/MeOH, Hx, IAC: FumoniPrep	HPLC-FD	10–20	42–75	[132]
Pig	Plasma, bile, urine, liver, kidney, muscle	1.5 mL 1 mL 1 mL 2 g 2.4 g	DON, DOM-1	LLE:Cl3, IAC: DON-test	LC-MS/MS	1.5–10	2–10	[137]
Goat	Plasma, urine, feces, liver	5 mL 5 g	ZAN and metabolites	LLE: EtOAc, IAC: Easi-Extract ZAN	HPLC	-	2.1–46.6	[135]
Bovine	Mucle, liver	5 g	ZON, α-ZOL, β-ZOL, ZAN, α-ZAL, β-ZAL	LLE: MeOH-Hx, SPE: Sep-pak amino	UPLC-MS/MS; CLEIA	0.5	0.5–0.7	[131]
Boar	Muscle, liver, kidney, spleen, cardiac muscle, lung, ovary, uterus	3 g	ZON, α-ZOL, β-ZOL	LLE: MeOH, IAC	LC-MS	1	-	[136]
Pig	Plasma, fat, muscle, stomach, brain, small intestines, heart, lung, spleen, urine, feces	0.5 mL 2 g	T-2, HT-2, T-2 triol	LLE: ACN. LLE: EtOAc, SPE: Varian Bond-Elut	LC-MS/MS	0.3–2	1–5	[126]
Broiler, poultry	Liver and meat	5 g	ENs, BEA	LLE, SPE	LC-MS/MS	0.015–0.56	0.03–1.12	[125]
Fish	Liver, viscera, tissue, head	5–10 g	ENs	LLE: ACN, SPE: C18	LC-MS/MS	0.3–3	1–10	[128]
Mice	Liver, kidney, colon, fat, brain, muscle, tumor urine, serum	0.2 g 50 μL	BEA, EN B	LLE: ACN	LC-MS/MS	-	0.05–0.15	[26]
Mice	Serum, Brain	50 μL	BEA, ENs	LLE: ACN-H_2_O	UPLC-MS/MS	0.3	-	[115]

**Table 7:** Studies of multi-mycotoxin determination in organs and tissues.

Species	Biological Sample	Sample Size	Mycotoxins	Extraction Procedure	Detection Technique	LOD (μg/kg)	LOQ (μg/kg)	Reference
Human	Urine and nasal secretions (nasal washes, sputa), heart, liver, urine		TCT, AFs, OTA	PBS, formalin	ELISA, Fluorometry	0.2–2	-	[114]
Human Rat Dogs Rabbit	*Human:* urine, blood, feces, saliva, nasal secretions, breast milk, amniotic fluid of pregnant women. *Animal:* liver, spleen, lung, kidney, stomach, colon, brain, urine, blood, and feces.	200 μL 200 μg	AFB1, AFB2, AFG1, AF2, AFM1, AFM2, OTA, DON, NIV, T-2, HT-2, 3-ADON, 15-ADON, NEO, FUS-X, DAS, MAS, ZON, ZAN, α-ZOL, β-ZOL, α-ZAL, β-ZAL, T-2 triol, T-2 tertraol, DOM-1, FB1, FB2	PLE: ACN/H_2_O/hx/acetic acid	HPLC-MS/MS	CCa: 0.01–0.69	0.2–0.5 CCb: 0.15–1.26	[28]
Chicken Pig	Muscle, liver	1g	T-2, HT-2, T-2-triol, NEO, DON, 3-Ac-DON, 5-Ac-DON, DOM, NIV	LLE: ACN/EtOAc SPE: Oasis HLB	UPLC-MS/MS	3–15	10–50	[129]
Rat	Plasma, liver, kidney	100 μL (25 mg tissue)	ABF1, OTA	LLE: CHCl_3_, IAC	UHPLC-FLD	0.01–0.3	2–8	[117]
Rat	Plasma, urine, liver, kidney, bladder, spleen, lung, stomach, small intestine, large intestine	-	DON-Glc, DON-GlcAs, ZON-14-Glc, ZON-14-GlcA, 3-ADON, 15-ADON	LLE: ACN	UPLC-MS/MS	0.3–16.3	0.6–54.4	[116]
Chicken	Muscle, liver, kidney, fat, tissues	2 g	DON, 3-ADON, 15-ADON, DOM-1	LLE: EtOAc, SPE: Oasis HLB	LC–MS/MS	CCa: 0.16–0.92	CCb: 0.68–2.07	[130]
Chickens	Muscle and liver	1 g	NIV, DON, DOM, NEO, 3-ADON, 15-ADON, T-2-triol, HT-2, T-2	LLE: ACN/H_2_O, SPE: Oasis HLB, IAC: charcoal/alumina/celite	UPLC-MS/MS	1–5	3–15	[129]
Broiler	Heart, liver, spleen, lung, kidney, Glandular stomach, muscular stomach, small intestine, muscle, bone, brain	1 g	T-2, HT-2, DAS	LLE: EtOAc	LC-MS/MS	0.02–0.05	0.08–0.17	[119]
Pig	Plasma, urine feces, liver, kidney, spleen, muscle, intestine, bile	1 mL 5 g	FUS-X, NIV	LLE: ACN/H_2_O, SPE: C18	LC-MS/MS	1.0–1.8	1.11–2.4	[124]
Pig	Bile, liver, and muscle	-	ZON, α-ZOL, β-ZOL, DON	LLE: MeOH/H_2_O, Hx, SPE: Oasis HLB	HPLC and EIA	-	-	[18]

CCa: decision limit; CCb: detection capability.

## References

[1] Turner N.W., Bramhmbhatt H., Szabo-Vezse M., Poma A., Coker R., Piletsky S.A. (2015). Analytical methods for determination of mycotoxins: An update (2009–2014). Anal. Chim. Acta.

[2] Tsitsigiannis I., Antoniou P., Tjamos C. (2012). Biological control strategies of mycotoxigenic fungi and associated mycotoxins in Mediterranean basin crops. Phytopathol. Mediterr..

[3] Xu L., Zhang Z., Zhang Q., Li P. (2016). Mycotoxin determination in foods using advanced sensors based on antibodies or aptamers. Toxins.

[4] Yang J., Li J., Jiang Y., Duan X., Qu H., Yang B., Chen F., Sivakumar D. (2014). Natural Occurrence, Analysis, and Prevention of Mycotoxins in Fruits and their Processed Products. Crit. Rev. Food Sci. Nutr..

[5] Zollner P., Mayer-Helm B. (2006). Trace mycotoxin analysis in complex biological and food matrices by liquid chromatography-atmospheric pressure ionisation mass spectrometry. J. Chromatogr. A.

[6] Escrivá L., Font G., Manyes L. (2015). In vivo toxicity studies of *Fusarium* mycotoxins in the last decade: A review. Food Chem. Toxicol..

[7] Guerre P. (2015). Fusariotoxins in Avian Species: Toxicokinetics, Metabolism and Persistence in Tissues. Toxins.

[8] Abrunhosa L., Morales H., Soares C., Calado T., Vila-Chã A.S., Pereira M., Venâncio A. (2016). A Review of Mycotoxins in Food and Feed Products in Portugal and Estimation of Probable Daily Intakes. Crit. Rev. Food Sci. Nutr..

[9] Moretti A., Susca A., Mulé G., Logrieco A.F., Proctor R.H. (2013). Molecular biodiversity of mycotoxigenic fungi that threaten food safety. Int. J. Food Microbiol..

[10] Summerell B.A., Leslie J.F. (2011). Fifty years of *Fusarium*: How could nine species have ever been enough?. Fungal Divers..

[11] Ismaiel A.A., Papenbrock J. (2015). Mycotoxins: Producing Fungi and Mechanisms of Phytotoxicity. Agriculture.

[12] Soto J.B., Ruiz M.J., Manyes L., Juan-García A. (2015). Blood, breast milk and urine: Potential biomarkers of exposure and estimated daily intake of ochratoxin A: A review. Food Addit. Contam. Part A.

[13] Xie L., Chen M., Ying Y. (2016). Development of Methods for Determination of Aflatoxins. Crit. Rev. Food Sci. Nutr..

[14] López P., Venema D., de Rijk T., de Kok A., Scholten J.M., Mol H.G.J., de Nijs M. (2016). Occurrence of *Alternaria* toxins in food products in The Netherlands. Food Control.

[15] Panel E., Chain F., European Food Safety Authority (2011). Scientific Opinion on the risks for animal and public health related to the presence of *Alternaria* toxins in feed and food. EFSA J..

[16] IARC Agents Classified by the IARC Monographs, Volumes 1–115. http://monographs.iarc.fr/ENG/Classification/List_of_Classifications_Vol1-115.pdf.

[17] Kwaśniewska K., Gadzała-Kopciuch R., Cendrowski K. (2015). Analytical Procedure for the Determination of Zearalenone in Environmental and Biological. Crit. Rev. Anal. Chem..

[18] Schneweis I., Meyer K., Ritzmann M., Hoffmann P., Dempfle L., Bauer J. (2005). Influence of organically or conventionally produced wheat on health, performance and mycotoxin residues in tissues and bile of growing pigs. Arch. Anim. Nutr..

[19] Amuzie C.J., Harkema J.R., Pestka J.J. (2008). Tissue distribution and proinflammatory cytokine induction by the trichothecene deoxynivalenol in the mouse: Comparison of nasal vs. oral exposure. Toxicology.

[20] Danicke S., Brezina U. (2013). Kinetics and metabolism of the *Fusarium* toxin deoxynivalenol in farm animals: Consequences for diagnosis of exposure and intoxication and carry over. Food Chem. Toxicol..

[21] Minervini F., Giannoccaro A., Nicassio M., Panzarini G., Lacalandra J.M. (2013). First Evidence of Placental Transfer of Ochratoxin A in Horses. Toxins.

[22] Britzi M., Friedman S., Miron J., Solomon R., Cuneah O., Shimshoni J.A., Soback S., Ashkenazi R., Armer S., Shlosberg A. (2013). Carry-Over of Aflatoxin B1 to Aflatoxin M1 in High Yielding Israeli Cows in Mid- and Late-Lactation. Toxins.

[23] Tozzi B., Liponi G.B., Meucci V., Casini L., Dall’Asta C., Intorre L., Gatta D. (2016). Aflatoxins M1 and M2 in the milk of donkeys fed with naturally contaminated diet. Dairy Sci. Technol..

[24] Winkler J., Kersten S., Valenta H., Meyer U., Engelhardt U.H., Dänicke S. (2015). Development of a multi-toxin method for investigating the carryover of zearalenone, deoxynivalenol and their metabolites into milk of dairy cows. Food Addit. Contam. Part A.

[25] Jonsson M., Jestoi M., Anthoni M., Welling A., Loivamaa I., Hallikainen V., Kankainen M., Lysøe E., Koivisto P., Peltonen K. (2016). *Fusarium* mycotoxin enniatin B: Cytotoxic effects and changes in gene expression profile. Toxicol. In Vitro.

[26] Rodríguez-Carrasco Y., Heilos D., Richter L., Süssmuth R.D., Heffeter P., Sulyok M., Kenner L., Berger W., Dornetshuber-Fleiss R. (2016). Mouse tissue distribution and persistence of the food-born fusariotoxins Enniatin B and Beauvericin. Toxicol. Lett..

[27] Armorini S., Al-Qudah K.M., Altafini A., Zaghini A., Roncada P. (2015). Biliary ochratoxin A as a biomarker of ochratoxin exposure in laying hens: An experimental study after administration of contaminated diets. Res. Vet. Sci..

[28] Cao X., Wu S., Yue Y., Wang S., Wang Y., Tao L., Tian H. (2013). A high-throughput method for the simultaneous determination of multiple mycotoxins in human and laboratory animal biological fluids and tissues by PLE and HPLC-MS/MS. J. Chromatogr. B.

[29] Serrano A.B., Font G., Mañes J., Ferrer E. (2016). Dispersive Liquid-Liquid Microextraction for the Determination of Emerging *Fusarium* Mycotoxins in Water. Food Anal. Meth..

[30] Song S., Ediage E.N., Wu A., De Saeger S. (2013). Development and application of salting-out assisted liquid/liquid extraction for multi-mycotoxin biomarkers analysis in pig urine with high performance liquid chromatography/tandem mass spectrometry. J. Chromatogr. A.

[31] Rodríguez-Carrasco Y., Mañes J., Berrada H., Font G. (2015). Preliminary Estimation of Deoxynivalenol Excretion through a 24 h Pilot Study. Toxins.

[32] Danicke S., Winkler J. (2015). Diagnosis of zearalenone (ZEN) exposure of farm animals and transfer of its residues into edible tissues (carry over). Food Chem. Toxicol..

[33] Suomela J.-P., Jarvinen R., Lassila M. (2010). Derivatization. First Dice Your Dill (Anethum graveolens L.) New Methods and Techniques in Sample Handling.

[34] Liu L.H., Zhou X.H., Shi H.C. (2015). Portable optical aptasensor for rapid detection of mycotoxin with a reversible ligand-grafted biosensing surface. Biosens. Bioelectron..

[35] Degen G.H., Mayer S., Blaszkewicz M. (2007). Biomonitoring of ochratoxin A in grain workers. Mycotoxin Res..

[36] Medina Á., Mateo E.M., Roig R.J., Blanquer A., Jiménez M. (2010). Ochratoxin A levels in the plasma of healthy blood donors from Valencia and estimation of exposure degree: Comparison with previous national Spanish data. Food Addit. Contam. Part A.

[37] Aslam M., Rivzi S.A.H., Beg A.E., Blaszkewicz M., Golka K., Degen G.H. (2012). Analysis of Ochratoxin a Blood Levels in Bladder Cancer Cases and Healthy Persons from Pakistan. J. Toxicol. Environ. Health Part A.

[38] Dohnal V., Dvorák V., Malír F., Ostry V., Roubal T. (2013). A comparison of ELISA and HPLC methods for determination of ochratoxin A in human blood serum in the Czech Republic. Food Chem. Toxicol..

[39] Koller G., Wichmann G., Rolle-Kampczyk U., Popp P., Herbarth O. (2016). Comparison of ELISA and capillary electrophoresis with laser-induced fluorescence detection in the analysis of Ochratoxin A in low volumes of human blood serum. J. Chromatogr. B.

[40] Kruger C.D., Cavaglieri L.R., Direito G.M., Keller K.M., Dalcero A.M., da Rocha Rosa C.A. (2010). Ochratoxin A in serum of swine from different Brazilian states. J. Vet. Diagn. Investig..

[41] Hmaissia Khlifaa K., Ghalib R., Mazigha C., Aounia Z., Machgoula S., Hedhili A. (2012). Ochratoxin A levels in human serum and foods from nephropathy patients in Tunisia: Where are you now?. Exp. Toxicol. Pathol..

[42] Sangare-Tigori B., Moukha S., Kouadio J.H., Dano D.S., Betbeder A.-M., Achour A., Creppy E.E. (2006). Ochratoxin A in human blood in Abidjan, Cote d’Ivoire. Toxicon.

[43] Malir F., Ostry V., Dofkova M., Roubal T., Dvorak V., Dohnal V. (2013). Ochratoxin A levels in blood serum of Czech women in the first trimester of pregnancy and its correspondence with dietary intake of the mycotoxin contaminant. Biomarkers.

[44] Muñoz K., Vega M., Rios G., Muñoz S., Madariaga R. (2006). Preliminary study of Ochratoxin A in human plasma in agricultural zones of Chile and its relation to food consumption. Food Chem. Toxicol..

[45] Fraeyman S., Devreese M., Antonissen G., De Baere S., Rychlik M., Croubels S. (2016). Comparative Oral Bioavailability, Toxicokinetics, and Biotransformation of Enniatin B1 and Enniatin B in Broiler Chickens. J. Agric. Food Chem..

[46] Devreese M., Osselaere A., Goossens J., Vandenbroucke V., De Baere S., Eeckhout M., De Backer P., Croubels S. (2012). New bolus models for in vivo efficacy testing of mycotoxin-detoxifying agents in relation to EFSA guidelines, assessed using deoxynivalenol in broiler chickens. Food Addit. Contam. Part A.

[47] Devreese M., Girgis G.N., Tran S.-T., De Baere S., De Backer P., Croubels S., Smith T.K. (2014). The effects of feed-borne *Fusarium* mycotoxins and glucomannan in turkey poults based on specific and non-specific parameters. Food Chem. Toxicol..

[48] Shin B.S., Hong S.H., Hwang S.W., Kim H.J., Lee J.B., Yoon H.-S., Kim D.J., Yoo S.D. (2009). Determination of zearalenone by liquid chromatography/tandem mass spectrometry and application to a pharmacokinetic study. Biomed. Chromatogr..

[49] Shin B.S., Hong S.H., Kim H.J., Yoon H.-S., Kim D.J., Hwang S.W., Lee J.B., Yoo S.D. (2009). Development of a Sensitive LC Assay with Fluorescence Detection for the Determination of Zearalenone in Rat Serum. Chromatographia.

[50] Saad-Hussein A., Taha M.M., Fadl N.N., Awad A.-H., Mahdy-Abdallah H., Moubarz G., Aziz H., El-Shamy K.A. (2016). Effects of airborne *Aspergillus* on serum aflatoxin B1 and liver enzymes in workers handling wheat flour. Hum. Exp. Toxicol..

[51] Devreese M., De Baere S., De Backer P., Croubels S. (2012). Quantitative determination of several toxicological important mycotoxins in pig plasma using multi-mycotoxin and analyte-specific high performance liquid chromatography–tandem mass spectrometric methods. J. Chromatogr. A.

[52] Devreese M., De Baere S., De Backer P., Croubels S. (2013). Quantitative determination of the *Fusarium* mycotoxins beauvericin, enniatin A, A1, B and B1 in pig plasma using high performance liquid chromatography–tandem mass spectrometry. Talanta.

[53] Devreese M., Broekaert N., De Mil T., Fraeyman S., De Backer P., Croubels S. (2014). Pilot toxicokinetic study and absolute oral bioavailability of the *Fusarium* mycotoxin enniatin B1 in pigs. Food Chem. Toxicol.

[54] Devreese M., Antonissen G., De Backer P., Croubels S. (2014). Efficacy of Active Carbon towards the Absorption of Deoxynivalenol in Pigs. Toxins.

[55] Devreese M., Antonissen G., Broekaert N., De Baere S., Vanhaecke L., De Backer P., Croubels S. (2015). Comparative Toxicokinetics, Absolute Oral Bioavailability, and Biotransformation of Zearalenone in Different Poultry Species. J. Agric. Food Chem..

[56] Broekaert N., Devreese M., De Mil T., Fraeyman S., De Baere S., De Saeger S., De Backer P., Croubels S. (2014). Development and validation of an LC-MS/MS method for the toxicokinetic study of deoxynivalenol and its acetylated derivatives in chicken and pig plasma. J. Chromatogr. B.

[57] De Baere S., Osselaere A., Devreese M., Vanhaecke L., De Backer P., Croubels S. (2012). Development of a liquid-chromatography tandem mass spectrometry and ultra-high-performance liquid chromatography high-resolution mass spectrometry method for the quantitative determination of zearalenone and its major metabolites in chicken and pig plasma. Anal. Chim. Acta.

[58] Ali N., Blaszkewicz M., Manirujjaman M., Perveen R., Al Nahid A., Mahmood S., Rahman M., Hossain K., Degen G.H. (2014). Biomonitoring of ochratoxin A in blood plasma and exposure assessment of adult students in Bangladesh. Mol. Nutr. Food Res..

[59] Tolosa J., Font G., Mañes J., Ferrer E. (2016). Multimycotoxin analysis in water and fish plasma by liquid chromatography-tandem mass spectrometry. Chemosphere.

[60] Pena A., Seifrtova M. (2006). Estimation of ochratoxin A in portuguese population: New data on the occurrence in human urine by high performance liquid chromatography with fluorescence detection. Food Chem. Toxicol..

[61] Manique R., Pena A., Lino C.M., Moltó J.C., Mañes J. (2008). Ochratoxin A in the morning and afternoon portions of urine from Coimbra and Valencian populations. Toxicon.

[62] Ali N., Muñoz K., Degen G.H. (2017). Ochratoxin A and its metabolites in urines of German adults—An assessment of variables in biomarker analysis. Toxicol. Lett..

[63] Vatinno R., Vuckovic D., Zambonin C.G., Pawliszyn J. (2008). Automated high-throughput method using solid-phase microextraction-liquid chromatography-tandem mass spectrometry for the determination of ochratoxin A in human urine. J. Chromatogr. A.

[64] Muñoz K., Cramer B., Dopstadt J., Humpf H.U., Degen G.H. (2017). Evidence of ochratoxin A conjugates in urine samples from infants and adults. Mycotoxin Res..

[65] Fushimi Y., Takagi M., Uno S., Kokushi E., Nakamura M., Hasunuma H., Shinya U., Deguchi E., Fink-Gremmels J. (2014). Measurement of Sterigmatocystin Concentrations in Urine for Monitoring the Contamination of Cattle Feed. Toxins.

[66] Warth B., Sulyok M., Berthiller F., Schuhmacher R., Fruhmann P., Hametner C., Adam G., Fröhlich J., Krska R. (2011). Direct quantification of deoxynivalenol glucuronide in human urine as biomarker of exposure to the *Fusarium* mycotoxin deoxynivalenol. Anal. Bioanal. Chem..

[67] Egner P.A., Groopman J.D., Wang J.-S., Kensler T.W., Friesen M.D. (2006). Quantification of Aflatoxin-B1-N7-Guanine in Human Urine by High-Performance Liquid Chromatography and Isotope Dilution Tandem Mass Spectrometry. Chem. Res. Toxicol..

[68] Gerding J., Cramer B., Humpf H. (2014). Determination of mycotoxin exposure in Germany using an LC-MS/MS multibiomarker approach. Mol. Nutr. Food Res..

[69] Gerding J., Ali N., Schwartzbord J., Cramer B., Brown D.L., Degen G.H., Humpf H. (2015). A comparative study of the human urinary mycotoxin excretion patterns in Bangladesh, Germany, and Haiti using a rapid and sensitive LC-MS/MS approach. Mycotoxin Res..

[70] Ezekiel C.N., Warth B., Ogara I.M., Abia W.A., Ezekiel V.C., Atehnkeng J., Sulyok M., Turner P.C., Tayo G.O., Krska R. (2014). Mycotoxin exposure in rural residents in northern Nigeria: A pilot study using multi-urinary biomarkers. Environ. Int..

[71] Warth B., Sulyok M., Fruhmann P., Mikula H., Berthiller F., Schuhmacher R., Hametner C., Abia W.A., Adam G., Fröhlich G. (2012). Development and validation of a rapid multi-biomarker liquid chromatography/tandem mass spectrometry method to assess human exposure to mycotoxins. Rapid Commun. Mass Spectrom..

[72] Warth B., Sulyok M., Fruhmann P., Berthiller F., Schuhmacher R., Hametner C., Adam G., Fröhlich J., Krska R. (2012). Assessment of human deoxynivalenol exposure using an LC-MS/MS based biomarker method. Toxicol. Lett..

[73] Heyndrickx E., Sioen I., Bellemans M., De Maeyer M., Callebaut A., De Henauw S., De Saeger S. (2014). Assessment of mycotoxin exposure in the Belgian population using biomarkers: Aim, design and methods of the BIOMYCO study. Food Addit. Contam. Part A.

[74] Heyndrickx E., Sioen I., Huybrechts B., Callebaut A., De Henauw S., De Saeger S. (2015). Human biomonitoring of multiple mycotoxins in the Belgian population: Results of the BIOMYCO study. Environ. Int..

[75] Andrés F., Zougagh M., Casta G., Ríos A. (2008). Determination of zearalenone and its metabolites in urine samples by liquid chromatography with electrochemical detection using a carbon nanotube-modified electrode. J. Chromatogr. A.

[76] Lattanzio V.M.T., Solfrizzo M., De Girolamo A., Chulze S.N., Torres A.M., Visconti A. (2011). LC-MS/MS characterization of the urinary excretion profile of the mycotoxin deoxynivalenol in human and rat. J. Chromatogr. B.

[77] Solfrizzo M., Gambacorta L., Visconti A. (2014). Assessment of Multi-Mycotoxin Exposure in Southern Italy by Urinary Multi-Biomarker Determination. Toxins.

[78] Blokland M.H., Sterk S.S., Stephany R.W., Launay F.M., Kennedy D.G., van Ginkel L.A. (2006). Determination of resorcylic acid lactones in biological samples by GC-MS. Discrimination between illegal use and contamination with *Fusarium* toxins. Anal. Bioanal. Chem..

[79] Ediage E.N., Di Mavungua J.D., Song S., Wu A., Van Peteghem C., De Saeger S. (2012). A direct assessment of mycotoxin biomarkers in human urine samples by liquid chromatography tandem mass spectrometry. Anal. Chim. Acta.

[80] Solfrizzo M., Gambacorta L., Lattanzio V.M.T., Powers S., Visconti A. (2011). Simultaneous LC-MS/MS determination of aflatoxin M1, ochratoxin A, deoxynivalenol, de-epoxydeoxynivalenol, α and β-zearalenols and fumonisin B1 in urine as a multi-biomarker method to assess exposure to mycotoxins. Anal. Bioanal. Chem..

[81] Gambacorta S., Solfrizzo H., Visconti A., Powers S., Cossalter A.M., Pinton P., Oswald I.P. (2013). Validation study on urinary biomarkers of exposure for aflatoxin B1, ochratoxin A, fumonisin B1, deoxynivalenol and zearalenone in piglets. World Mycotoxin J..

[82] Ahn J., Kim D., Kim H., Jahng K.-Y. (2010). Quantitative determination of mycotoxins in urine by LC-MS/MS. Food Addit. Contam. Part A.

[83] Rubert J., Soriano J.M., Mañes J., Soler C. (2011). Rapid mycotoxin analysis in human urine: A pilot study. Food Chem. Toxicol..

[84] Turner P.C., Ji B.T., Shu X.O., Zheng W., Chow W.-H., Gao Y.T., Hardie L.J. (2011). A biomarker survey of urinary deoxynivalenol in China: The Shanghai Women’s Health Study. Food Addit. Contam. Part A Chem. Anal. Control Expo. Risk Assess..

[85] Ali N., Blaszkewicz M., Al Nahid A., Rahman M., Degen G.H. (2015). Deoxynivalenol Exposure Assessment for Pregnant Women in Bangladesh. Toxins.

[86] Ali N., Blaszkewicz M., Degen G.H. (2016). Assessment of deoxynivalenol exposure among Bangladeshi and German adults by a biomarker-based approach. Toxicol. Lett..

[87] Ali N., Blaszkewicz M., Degen G.H. (2015). Occurrence of the mycotoxin citrinin and its metabolite dihydrocitrinone in urines of German adults. Arch. Toxicol..

[88] Ali N., Blaszkewicz M., Mohanto N.C., Rahman M., Alim A., Hossain K., Degen G.H. (2015). First results on citrinin biomarkers in urines from rural and urban cohorts in Bangladesh. Mycotoxin Res..

[89] Silva L.J.G., Pena A., Lino C.M., Fernández M.F., Mañes J. (2010). Fumonisins determination in urine by LC-MS-MS. Anal. Bioanal. Chem..

[90] Dusi G., Bozzoni E., Assini W., Tognoli N., Gasparini M., Ferretti E. (2009). Confirmatory method for the determination of resorcylic acid lactones in urine sample using immunoaffinity cleanup and liquid chromatography-tandem mass spectrometry. Anal. Chim. Acta.

[91] Rejczak T., Tuzimski T. (2015). A review of recent developments and trends in the QuEChERS sample preparation approach. Open Chem..

[92] Rodríguez-Carrasco Y., Moltó J.C., Mañes J., Berrada H. (2014). Development of a GC–MS/MS strategy to determine 15 mycotoxins and metabolites in human urine. Talanta.

[93] Rodríguez-Carrasco Y., Moltó J.C., Mañes J., Berrada H. (2014). Exposure assessment approach through mycotoxin/creatinine ratio evaluation in urine by GC-MS/MS. Food Chem. Toxicol..

[94] Rodríguez-Carrasco Y., Moltó J.C., Mañes J., Berrada H. (2017). Development of microextraction techniques in combination with GC-MS/MS for the determination of mycotoxins and metabolites in human urine. J. Sep. Sci..

[95] Muñoz K., Blaszkewicz M., Degen G.H. (2010). Simultaneous analysis of ochratoxin A and its major metabolite ochratoxin alpha in plasma and urine for an advanced biomonitoring of the mycotoxin. J. Chromatogr. B.

[96] Ali N., Blaszkewicz M., Manirujjaman M., Degen G.H. (2016). Biomonitoring of concurrent exposure to ochratoxin A and citrinin in pregnant women in Bangladesh. Mycotoxin Res..

[97] Brezina U., Rempe I., Kersten S., Valenta H., Humpf H.-U., Dänicke S. (2014). Diagnosis of intoxications of piglets fed with *Fusarium* toxin-contaminated maize by the analysis of mycotoxin residues in serum, liquor and urine with LC-MS/MS. Arch. Anim. Nutr..

[98] Serrano A.B., Capriotti A.L., Cavaliere C., Piovesana S., Samperi R., Ventura S., Laganà A. (2015). Development of a Rapid LC-MS/MS Method for the Determination of Emerging *Fusarium* mycotoxins Enniatins and Beauvericin in Human Biological Fluids. Toxins.

[99] Blaszkewicz M., Muñoz K., Degen G.H. (2013). Methods for analysis of citrinin in human blood and urine. Arch. Toxicol..

[100] Escrivá L., Font G., Manyes L. (2015). Quantitation of enniatins in biological samples of Wistar rats after oral administration by LC-MS/MS. Toxicol. Mech. Methods.

[101] Juan C., Manyes L., Font G., Juan-Garcia A. (2014). Evaluation of immunologic effect of Enniatin A and quantitative determination in feces, urine and serum on treated Wistar rats. Toxicon.

[102] Songsermsakul P., Sontag G., Cichna-Markl M., Zentek J., Razzazi-Fazeli E. (2006). Determination of zearalenone and its metabolites in urine, plasma and faeces of horses by HPLC-APCI-MS. J. Chromatogr. B.

[103] Schwartz-Zimmermann H.E., Fruhmann P., Dänicke S., Wiesenberger G., Caha S., Weber J., Berthiller F. (2015). Metabolism of Deoxynivalenol and Deepoxy-Deoxynivalenol in Broiler Chickens, Pullets, Roosters and Turkeys. Toxins.

[104] Warth B., Sulyok M., Krska R. (2013). LC-MS/MS-based multibiomarker approaches for the assessment of human exposure to mycotoxins. Anal. Bioanal. Chem..

[105] Huybrechts B., Martins J.C., Debongnie P., Uhlig S., Callebaut A. (2015). Fast and sensitive LC-MS/MS method measuring human mycotoxin exposure using biomarkers in urine. Arch. Toxicol..

[106] Gürbay A., Girgin G., Sabuncuog S.A.Ş., Şahin G., Yurdakök M., Yig Ş., Tekinalp G. (2009). Ochratoxin A: Is it present in breast milk samples obtained from mothers from Ankara, Turkey?. J. Appl. Toxicol..

[107] Afshar P., Shokrzadeh M., Kalhori S., Babaee Z., Saravi S.S.S. (2013). Occurrence of Ochratoxin A and A flatoxin M1 in human breast milk in Sari, Iran. Food Control.

[108] Camel V., Ouethrani M., Coudray C., Philippe C., Rabot S. (2012). Semi-automated solid-phase extraction method for studying the biodegradation of ochratoxin A by human intestinal microbiota. J. Chromatogr. B.

[109] Andrade P.D., Gomes da Silva J.L., Caldas E.D. (2013). Simultaneous analysis of aflatoxins B1, B2, G1, G2, M1 and ochratoxinA in breast milk by high-performance liquidchromatography/fluorescence after liquid-liquid extraction with lowtemperature purification (LLE-LTP). J. Chromatogr. A.

[110] Rubert J., León N., Sáez C., Martins C.P.B., Godula M., Yusà V., Mañes J., Soriano J.M., Soler C. (2014). Evaluation of mycotoxins and their metabolites in human breast milk using liquid chromatography coupled to high resolution mass spectrometry. Anal. Chim. Acta.

[111] Massart F., Micillo F., Rivezzi G., Perrone L., Baggiani A., Miccoli M., Meucci V. (2016). Zearalenone screening of human breast milk from the Naples area. Toxicol. Environ. Chem..

[112] De Baere S., Goossens J., Osselaere A., Devreese M., Vandenbroucke V., De Backer P., Croubels S. (2011). Quantitative determination of T-2 toxin, HT-2 toxin, deoxynivalenol and deepoxy-deoxynivalenol in animal body fluids using LC-MS/MS detection. J. Chromatogr. B.

[113] Osselaere A., Devreese M., Goossens J., Vandenbroucke V., De Baere S., De Backer P., Croubels S. (2013). Toxicokinetic study and absolute oral bioavailability of deoxynivalenol, T-2 toxin and zearalenone in broiler chickens. Food Chem. Toxicol..

[114] Hooper D.G., Bolton V.E., Guilford F.T., Straus D.C. (2009). Mycotoxin Detection in Human Samples from Patients Exposed to Environmental Molds. Int. J. Mol. Sci..

[115] Taevernier L., Bracke N., Veryser L., Wynendaele E., Gevaert B., Peremans K., De Spiegeleer B. (2016). Blood-brain barrier transport kinetics of the cyclic depsipeptide mycotoxins beauvericin and enniatins. Toxicol. Lett..

[116] Veršilovskis A., Geys J., Huybrechts B., Goossens E., De Saeger S., Callebaut A. (2012). Simultaneous determination of masked forms of deoxynivalenol and zearalenone after oral dosing in rats by LC-MS/MS. World Mycotoxin J..

[117] Corcuera L., Ibáñez-Vea M., Vettorazzi A., González-Peñas E., López de Cerain A. (2011). Validation of a UHPLC-FLD analytical method for the simultaneous quantification of aflatoxin B1 and ochratoxin a in rat plasma, liver and kidney. J. Chromatogr. B.

[118] Manyes L., Escriva L., Belen Serrano A., Rodriguez-Carrasco Y., Tolosa J., Meca G., Font G. (2014). A preliminary study in Wistar rats with enniatin A contaminated feed. Toxicol. Mech. Methods.

[119] Yang L., Zhao Z., Wu A., Deng Y., Zhou Z. (2013). Determination of trichothecenes A (T-2 toxin, HT-2 toxin, and diacetoxyscirpenol) in the tissues of broilers using liquid chromatography coupled to tandem mass spectrometry. J. Chromatogr. B.

[120] Milicevic D., Juric V., Stefanovic S., Baltic T., Jankovic S. (2010). Evaluation and Validation of Two Chromatographic Methods (HPLC-Fluorescence and LC-MS/MS) for the Determination and Confirmation of Ochratoxin A in Pig Tissues. Arch. Environ. Contam. Toxicol..

[121] Vettorazzi A., Gonzalez-Peñas E., Arbillagaa L., Corcuera L.-A., López de Ceraina A. (2008). Simple high-performance liquid chromatography-fluorescence detection method for plasma, kidney and liver of rat as a tool for toxicology studies. J. Chromatogr. A.

[122] Shin B.S., Hong S.H., Bulitta J.B., Hwang S.W., Kim H.J., Lee J.B., Yang S.D., Kim J.E., Yoon H.S., Kim D.J. (2009). Disposition, Oral Bioavailability, and Tissue Distribution of Zearalenone in Rats at Various Dose Levels Disposition. J. Toxicol. Environ. Health Part A.

[123] Chandratre G.A., Telang A.G., Badgujar P.C., Raut S.S., Sharma A.K. (2014). Toxicopathological Alterations Induced by High Dose Dietary T-2 Mycotoxin and its Residue Detection in Wistar Rats. Arch. Environ. Contam. Toxicol..

[124] Saengtienchai T., Poapolathep S., Isariyodom S., Ikenaka Y., Ishizuka M., Poapolathep A. (2014). Toxicokinetics and tissue depletion of Fusarenon-X and its metabolite nivalenol in piglets. Food Chem. Toxicol..

[125] Kongkapan J., Giorgi M., Poapolathep S., Isariyodom S., Poapolathep A. (2016). Toxicokinetics and tissue distribution of nivalenol in broiler chickens. Toxicon.

[126] Sun Y., Zhang G., Zhao H., Zheng J., Hu F., Fang B. (2014). Liquid chromatography-tandem mass spectrometry method for toxicokinetics, tissue distribution, and excretion studies of T-2 toxin and its major metabolites in pigs. J. Chromatogr. B.

[127] Jestoi M., Rokka M., Peltonen K. (2007). An integrated sample preparation to determine coccidiostats and emerging *Fusarium*-mycotoxins in various poultry tissues with LC-MS/MS. Mol. Nutr. Food Res..

[128] Tolosa J., Font G., Mañes J., Ferrer E. (2014). Natural Occurrence of Emerging *Fusarium* Mycotoxins in Feed and Fish from Aquaculture. J. Agric. Food Chem..

[129] Yang S., Wang Y., Beier R.C., Zhang H., De Ruyck K., Sun F., Cao X., Shen J., Zhang D., Wang Z. (2015). Simultaneous Determination of Type A and B Trichothecenes and Their Main Metabolites in Food Animal Tissues by Ultraperformance Liquid Chromatography Coupled with Triple-Quadrupole Mass Spectrometry. J. Agric. Food Chem..

[130] Xu L., Zhang G., Guo C., Zhang Y., Zhang Y., Zheng J., Yang H., Yang D., He L., Zeng Z. (2014). Simultaneous determination of major type-B trichothecenes and the de-epoxy metabolite of deoxynivalenol in chicken tissues by HPLC-MS/MS. J. Sep. Sci..

[131] Haiyang J., Wenjun W., Jinghui Z., Xiaoqi T., Jiancheng L., Xi X., Kai W., Fei X., Zhaopeng W., Min C. (2014). Determination of zeranol and its metabolites in bovine muscle and liver by a chemiluminescence enzyme immunoassay: Compared to an ultraperformance liquid chromatography tandem mass spectroscopy method. Luminescence.

[132] Pagliuca G., Zironi E., Ceccolini A., Matera R., Paolo G., Piva A. (2005). Simple method for the simultaneous isolation and determination of fumonisin B1 and its metabolite aminopentol-1 in swine liver by liquid chromatography-fluorescence detection. J. Chromatogr. B.

[133] Hashimoto Y., Katsunuma Y., Nunokawa M., Minato H., Yonemochi C. (2016). Influence of repeated ochratoxin A ingestion on milk production and its carry-over into the milk, blood and tissues of lactating cows. Anim. Sci. J..

[134] Wiśniewska-Dmytrow H., Żmudzki J., Burek O., Pietruszka K. (2013). Official control of ochratoxin A in food of animal origin in Poland between 2003 and 2012. J. Nat. Vet. Res. Inst. Pulawy.

[135] Dong M., He X.J., Tulayakul P., Li J.-Y., Dong K.-S., Manabe N., Nakayama H., Kumagai S. (2010). The toxic effects and fate of intravenously administered zearalenone in goats. Toxicon.

[136] Gajecka M., Sławuta P., Nicpon J., Kołacz R., Kiełbowicz Z., Zielonka L., Dąbrowski M., Szweda W., Gajecki M., Nicpon J. (2016). Zearalenone and its metabolites in the tissues of female wild boars exposed per os to mycotoxins. Toxicon.

[137] Danicke S., Beyer M., Breves G., Valenta H., Humpf H.-U. (2010). Effects of oral exposure of pigs to deoxynivalenol (DON) sulfonate (DONS) as the non-toxic derivative of DON on tissue residues of DON and de-epoxy-DON and on DONS blood levels. Food Addit. Contam. Part A.

[138] Tardieu D., Bailly J., Skiba F., Grosjean F., Guerre P. (2008). Toxicokinetics of fumonisin B1 in turkey poults and tissue persistence after exposure to a diet containing the maximum European tolerance for fumonisins in avian feeds. Food Chem. Toxicol..

[139] Pestka J.J., Islam Z., Amuzie C.J. (2008). Immunochemical assessment of deoxynivalenol tissue distribution following oral exposure in the mouse. Toxicol. Lett..

[140] Mally A., Solfrizzo M., Degen G.H. (2016). Biomonitoring of the mycotoxin Zearalenone: Current state-of-the art and application to human exposure assessment. Arch. Toxicol..

[141] Ediage E.N., Diana J., Mavungu D., Song S., Sioen I., De Saeger S. (2013). Multimycotoxin analysis in urines to assess infant exposure: A case study in Cameroon. Environ. Int..

[142] Zollner P., Jodlbauer J., Kleinova M., Kahlbacher H., Kuhn T., Hochsteiner W., Lindner W. (2002). Concentration Levels of Zearalenone and Its Metabolites in Urine, Muscle Tissue, and Liver Samples of Pigs Fed with Mycotoxin-Contaminated Oats. J. Agric. Food Chem..

[143] European Food Safety Authority (2006). Opinion of the scientific panel on contaminants in the food chain on a request from the commission related to ochratoxin A in food. EFSA J..

[144] European Commission (2002). Assessment of Dietary Intake of Ochratoxin A by the Population of European Union Members States. Directorate General-Health and Consumer Protection. Report on Tasks for Scientific Cooperation. Report of Experts Participating in Task 3.2.7.

[145] European Food Safety Authority (2012). Panel on Contaminants in the Food Chain. Scientific opinion on the risks for public and animal health related to the presence of citrinin in food and feed. EFSA J..

[146] Scientific Committee on Food (SCF) (2003). Updated Opinion of the Scientific Committee on Food on Fumonisin B1, B2 and B3: SCF/CS/CNTM/MYC/28 Final.

[147] Warth B., Braun D., Ezekiel C.N., Turner P.C., Degen G.H., Marko D. (2016). Biomonitoring of Mycotoxins in Human Breast Milk: Current State and Future Perspectives. Chem. Res. Toxicol..

[148] Kolossova A., Stroka J., Breidbach A., Kroeger K., Ambrosio M., Bouten K., Ulberth F. (2009). Evaluation of the Effect of Mycotoxin Binders in Animal Feed on the Analytical Performance of Standardised Methods for the Determination of Mycotoxins in Feed.

[149] Jacela J.Y., De Rouchey J.M., Tokach M.D., Goodband R.D., Nelssen J.L., Renter D.G., Dritz S.S. (2010). Feed additives for swine: Fact sheets-flavors and mold inhibitors, mycotoxin binders, and antioxidants. J. Swine Health Prod..

